# Plant‐derived Durvalumab variants show efficient PD‐1/PD‐L1 blockade and therapeutically favourable FcR binding

**DOI:** 10.1111/pbi.14260

**Published:** 2023-12-04

**Authors:** Shiva Izadi, Simon Gumpelmair, Pedro Coelho, Henrique O. Duarte, Joana Gomes, Judith Leitner, Vinny Kunnummel, Lukas Mach, Celso A. Reis, Peter Steinberger, Alexandra Castilho

**Affiliations:** ^1^ Department of Applied Genetics and Cell Biology Institute for Plant Biotechnology and Cell Biology, University of Natural Resources and Life Sciences Vienna Austria; ^2^ Division of Immune Receptors and T Cell Activation Institute of Immunology, Center for Pathophysiology, Infectiology and Immunology, Medical University of Vienna Vienna Austria; ^3^ i3S – Instituto de Investigação e Inovação em Saúde, Universidade do Porto Porto Portugal; ^4^ Institute of Molecular Pathology and Immunology of the University of Porto (IPATIMUP) Porto Portugal; ^5^ Instituto de Ciências Biomédicas Abel Salazar (ICBAS), Universidade do Porto Porto Portugal; ^6^ Faculty of Medicine (FMUP) University of Porto Porto Portugal

**Keywords:** Durvalumab, checkpoint inhibitors, PD‐1/PD‐L1, FcγR, FcRn, *Nicotiana benthamiana*

## Abstract

Immune checkpoint blocking therapy targeting the PD‐1/PD‐L1 inhibitory signalling pathway has produced encouraging results in the treatment of a variety of cancers. Durvalumab (Imfinzi^®^) targeting PD‐L1 is currently used for immunotherapy of several tumour malignancies. The Fc region of this IgG1 antibody has been engineered to reduce FcγR interactions with the aim of enhancing blockade of PD‐1/PD‐L1 interactions without the depletion of PD‐L1‐expressing immune cells. Here, we used *Nicotiana benthamiana* to produce four variants of Durvalumab (DL): wild‐type IgG1 and its ‘Fc‐effector‐silent’ variant (LALAPG) carrying further modifications to increase antibody half‐life (YTE); IgG4_S228P_ and its variant (PVA) with Fc mutations to decrease binding to FcγRI. In addition, DL variants were produced with two distinct glycosylation profiles: afucosylated and decorated with α1,6‐core fucose. Plant‐derived DL variants were compared to the therapeutic antibody regarding their ability to (i) bind to PD‐L1, (ii) block PD‐1/PD‐L1 inhibitory signalling and (iii) engage with the neonatal Fc receptor (FcRn) and various Fcγ receptors. It was found that plant‐derived DL variants bind to recombinant PD‐L1 and to PD‐L1 expressed in gastrointestinal cancer cells and are able to effectively block its interaction with PD‐1 on T cells, thereby enhancing their activation. Furthermore, we show a positive impact of Fc amino acid mutations and core fucosylation on DL's therapeutic potential. Compared to Imfinzi^®^, DL‐IgG1 (LALAPG) and DL‐IgG4 (PVA)_S228P_ show lower affinity to CD32B inhibitory receptor which can be therapeutically favourable. Importantly, DL‐IgG1 (LALAPG) also shows enhanced binding to FcRn, a key determinant of serum half‐life of IgGs.

## Introduction

Immunological checkpoint inhibitors have emerged as novel therapy for cancer treatment (Li *et al*., 2018). Immune checkpoints are an essential part of the immune system and represent a group of membrane proteins expressed on effector cells, composed of multiple co‐inhibitory and co‐stimulatory pathways (Chen and Flies, 2013; Croft, 2003). Programmed cell death 1 (PD‐1) is one of the most effective T‐cell immune checkpoint molecules (Fife and Bluestone, 2008). PD‐1 is a transmembrane protein receptor expressed on the surface of a variety of immune cells, including monocytes, T and B cells, acting as an ‘off switch’ to prevent excessive inflammation and maintaining immune tolerance to self‐antigens under normal conditions (Keir *et al*., 2008). It does this when it attaches to its ligands, programmed cell death ligand PD‐L1 and PD‐L2, expressed on a variety of cells, including antigen‐presenting cells (Keir *et al*., 2008). Engagement of PD‐1 by PD‐L1 transmits inhibitory signals that suppress T‐cell function which can be reversed once signalling through PD‐1 ceases. These inhibitory mechanisms should prevent aberrant immune responses and protect healthy tissues. However, by overexpressing PD‐L1, tumour cells take advantage of this pathway to escape immune surveillance (Han *et al*., 2020; Zou and Chen, 2008).

PD‐L1 is up‐regulated in the tumour microenvironment and is found in a large variety of tumour cells. Therefore, immune checkpoint blockade can be a highly effective therapeutic strategy (Constantinidou *et al*., 2019; Iwai *et al*., 2017; Ribas and Wolchok, 2018). PD‐L1 expression in tumours is currently used as a biomarker to inform therapeutic decisions. Monoclonal antibodies (mAbs) acting as PD‐1 and PD‐L1 inhibitors are a group of immunotherapy drugs that block the interaction between PD‐1 and PD‐L1 preventing the ‘off’ signal from being transmitted and, therefore, boosting T‐cell immunity against a variety of human cancers (Gong *et al*., 2018; Waldman *et al*., 2020). Therapeutic antibodies used for the treatment of cancer patients normally belong to the IgG1 subclass and induce tumour destruction via the recruitment of immune effector cells by the Fc and hinge regions. However, antibodies have different requirements for Fcγ receptor engagement to attain optimal anti‐tumour activity (Yu *et al*., 2020). For blocking antibodies, such as the ones targeting PD‐1 and PD‐L1 which are not tumour‐specific, the cytotoxicity brought about by antibody‐dependent cellular cytotoxicity and phagocytosis (ADCC/ADCP) should be averted (Boulard *et al*., 2022).

To date, the FDA has approved six mAbs targeting PD‐1 (Nivolumab, Pembrolizumab and Cemiplimab) and PD‐L1 (Atezolizumab, Durvalumab and Avelumab) for the treatment of haematological and solid malignancies, and many others are currently under development (Ai *et al*., 2020; Boulard *et al*., 2022; Chen *et al*., 2021; Vaddepally *et al*., 2020).

A comparative study of FDA‐approved antibodies targeting the PD‐1/PD‐L1 axis showed that PD‐L1 antibodies seem to be superior to PD‐1 antibodies in reverting PD‐1 signalling (De Sousa Linhares *et al*., 2019).

All current FDA‐approved PD‐L1 antibodies are from the IgG1 isotype. Atezolizumab is engineered with a mutation of N297 to prevent *N*‐glycosylation and thus exhibits decreased FcγR‐binding and effector functions (Herbst *et al*., 2014). However, the pronounced aggregation tendency of the non‐glycosylated protein compromises the therapeutic efficacy of Atezolizumab. Durvalumab (DL) is engineered to contain amino acid substitutions that reduce Fc‐mediated effector functions against cells expressing PD‐L1 (Oganesyan *et al*., 2008; Stewart *et al*., 2015) while Avelumab retains intact Fc functions and can induce ADCC as a part of its mechanism of action (Hamilton and Rath, 2017). However, despite exerting enhanced cytotoxic effects towards PD‐L1‐expressing tumour cells, the effector function can diminish antitumor responses by attacking activated effector T cells and other immune cells, which also express high levels of PD‐L1 (Knorr and Ravetch, 2019; Leitner *et al*., 2021).

PD‐L1 is heavily glycosylated and glycosylation affects its binding to PD‐1 and to diagnostic antibodies leading to inaccurate readouts (Lee *et al*., 2019). Removal of PD‐L1 *N*‐linked glycosylation by enzymatic digestion eliminates structural hindrance and improves antibody‐based detection and binding of Atezolizumab in lung cancer cells (Lee *et al*., 2019). In contrast, for breast cancer cells, binding of all three therapeutic PD‐L1 antibodies favoured glycosylated PD‐L1 over the non‐glycosylated protein, and DL showed the highest affinity (Benicky *et al*., 2021).

Traditionally, therapeutic mAbs are manufactured in mammalian cell factories. Recently, plant‐based expression platforms have emerged as cost‐effective alternatives to produce high‐quality proteins for research, diagnostic and therapeutic applications. Many plant‐made pharmaceuticals have entered clinical testing, including antibodies for the treatment of cancer (Nessa *et al*., 2020; Shanmugaraj *et al*., 2020). In addition, advances in plant engineering have resulted in the ability to produce antibodies with tailor‐made glycans (Castilho *et al*., 2015). mAbs produced in plants differ only in Fc *N*‐glycosylation when compared to those produced in mammalian cells. When expressed in wild‐type plants, mAbs are decorated with complex glycans carrying plant‐specific glycosylation (core α1,3‐fucose and α1,2‐xylose). These sugar residues do not appear to affect the safety and efficacy of the mAbs but they are considered as cross‐reactive carbohydrate determinants (CCDs) (Platts‐Mills *et al*., 2021).

Here, we have used a glycoengineered *Nicotiana benthamiana* (*N. benthamiana*) plant line (ΔXF) (Strasser *et al*., 2008) devoid of α1,3‐fucosylation and β1,2‐xylosylation to produce DL in four heavy chain (HC) variants: wild‐type IgG1 and wild‐type IgG4_S228P_; Fc‐silent IgG1 and Fc‐silent IgG4_S228P_. In addition, DL variants were produced with two different glycosylation profiles, afucosylated and human‐like α1,6‐core‐fucosylated. The plant‐derived DL variants were then compared to clinical‐grade DL (Imfinzi^®^, AstraZeneca) in terms of their ability to (i) bind to recombinant PD‐L1 and to PD‐L1 overexpressed in gastrointestinal cancer cells, (ii) block PD‐1/PD‐L1 interactions in cell‐based assays and (iii) engage with activating (CD16, CD32A and CD64), inhibitory (CD32B) and neonatal (FcRn) Fc receptors.

## Results

### Generation of Durvalumab HC‐variants

Durvalumab (Imfinzi^®^) is a fully human IgG1 kappa antibody produced in Chinese hamster ovary (CHO) *cells* and engineered to contain three amino acid substitutions in the hinge region (L234F/L235E/P331S) to reduce FcγR binding and ADCC or CDC cytotoxicity (Oganesyan *et al*., 2008; Stewart *et al*., 2015). The Fc domain is glycosylated with mainly biantennary complex‐type *N*‐glycans.

**Figure 1:**
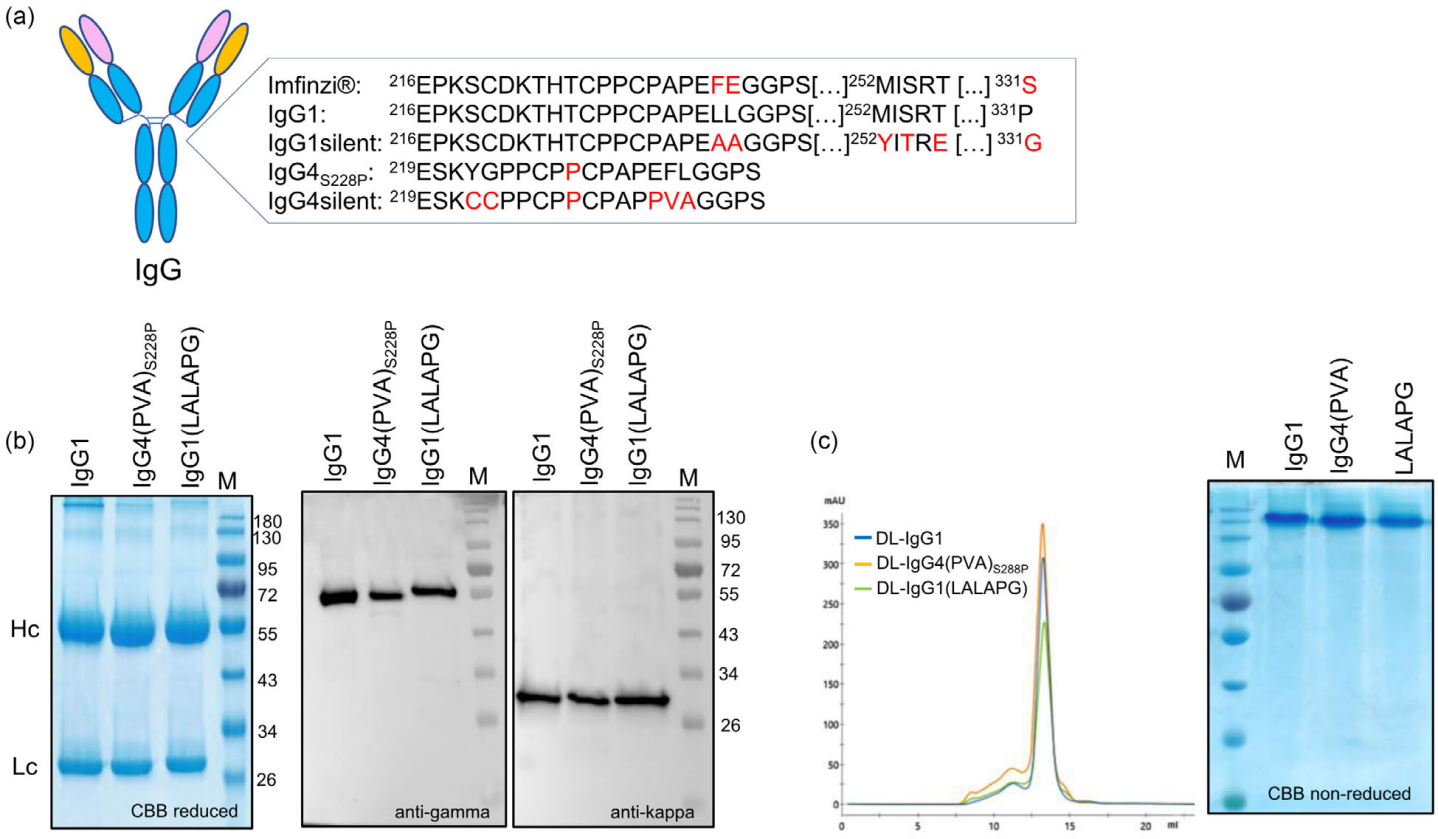
Expression and purification of plant‐derived Durvalumab variants. (a) Schematic representation of an IgG highlighting the differences in amino acid sequence within the hinge and CH2 domain of Durvalumab (Imfinzi^®^) and plant‐derived DL variants. (b) DL variants expressed in *N. benthamiana* ΔXF plants and purified by affinity chromatography were analysed in reducing conditions. SDS‐PAGE gels were either stained with Coomassie brilliant blue (CBB) or used for immunoblotting with anti‐gamma chain (heavy chain) and anti‐kappa chain (light chain) antibodies conjugated to HRP. The apparent molecular mass of marker proteins is shown in kilo Dalton (kDa). (c) Size exclusion chromatography profiles and CBB of SDS‐PAGE gels run under non‐reducing conditions.

Durvalumab (DL) was first expressed in *N. benthamiana* as four variants with the same variable regions but differing in their hinge‐constant region of the γ‐chain (Figure 1a). Besides the wild‐type DL‐IgG1 and DL‐IgG4_S228P_, a Fc‐silent IgG1 variant was generated by introducing two triple mutations: L234A/L235A/P331G (LALAPG) to decrease binding to human CD64, CD32A, CD16 and C1q (Schlothauer *et al*., 2016) and M252Y/S254T/T256E (YTE) known to prolong half‐life and increase mAb bioavailability (Dall'Acqua *et al*., 2002).

The completely Fc‐silent DL‐IgG4_S228P_ carries a combination of six mutations in the hinge region: S228P to prevent half‐mAb formation and Fab‐arm exchange (Silva *et al*., 2015); Y219C and G220C to increase the stability of the heavy–heavy chain interaction via incorporation of additional disulphide bonds (Handlogten *et al*., 2020); and E233P/F234V/P235A (PVA) to reduce the ability to bind to CD64 (Zhang *et al*., 2018). All DL HC variants carry the conserved glycosylation site (N297) in the CH2 domain known to impact their binding affinity to FcγRs (Arnold *et al*., 2007).

DL variants were transiently expressed in *N. benthamiana*, a tobacco‐related plant species widely used for recombinant protein expression, using a viral‐based vector expression system developed by Icon Genetics (magnICON) (Castilho *et al*., 2015; Klimyuk *et al*., 2014).

### Expression and glycoengineering of Durvalumab HC variants

Tobacco mosaic virus‐based (TMVα) vectors carrying the DL HC variants were mixed with the potato *virus* X‐based (PVXα)‐DL‐LC (1 : 1) and co‐expressed in the *N. benthamiana* glycosylation mutant ∆XF (Strasser *et al*., 2008) via agroinfiltration. Total soluble proteins extracted after 4 days postinfiltration (4 dpi) were used to purify the antibodies via affinity chromatography with protein A. The quality and purity of plant‐derived DL were confirmed by SDS‐PAGE. Coomassie brilliant blue (CBB) staining of SDS‐PAGE gels run under reducing conditions showed two bands representing the heavy and light chains which was confirmed by Western blotting using anti‐human gamma and anti‐human kappa antibodies (Figure 1b). Both chains seem to be stable *in planta* with no significant degradation observed. Size exclusion chromatography (SEC) showed similar profiles for all DL variants with a major dimer peak (>80%) indicating that the majority of the protein is correctly assembled (Figure 1c). The overall yield after protein A purification (measured by UV absorbance) was similar for all DL variants (range: 0.6–1.0 g/kg leaf wet weight).

**Figure 2:**
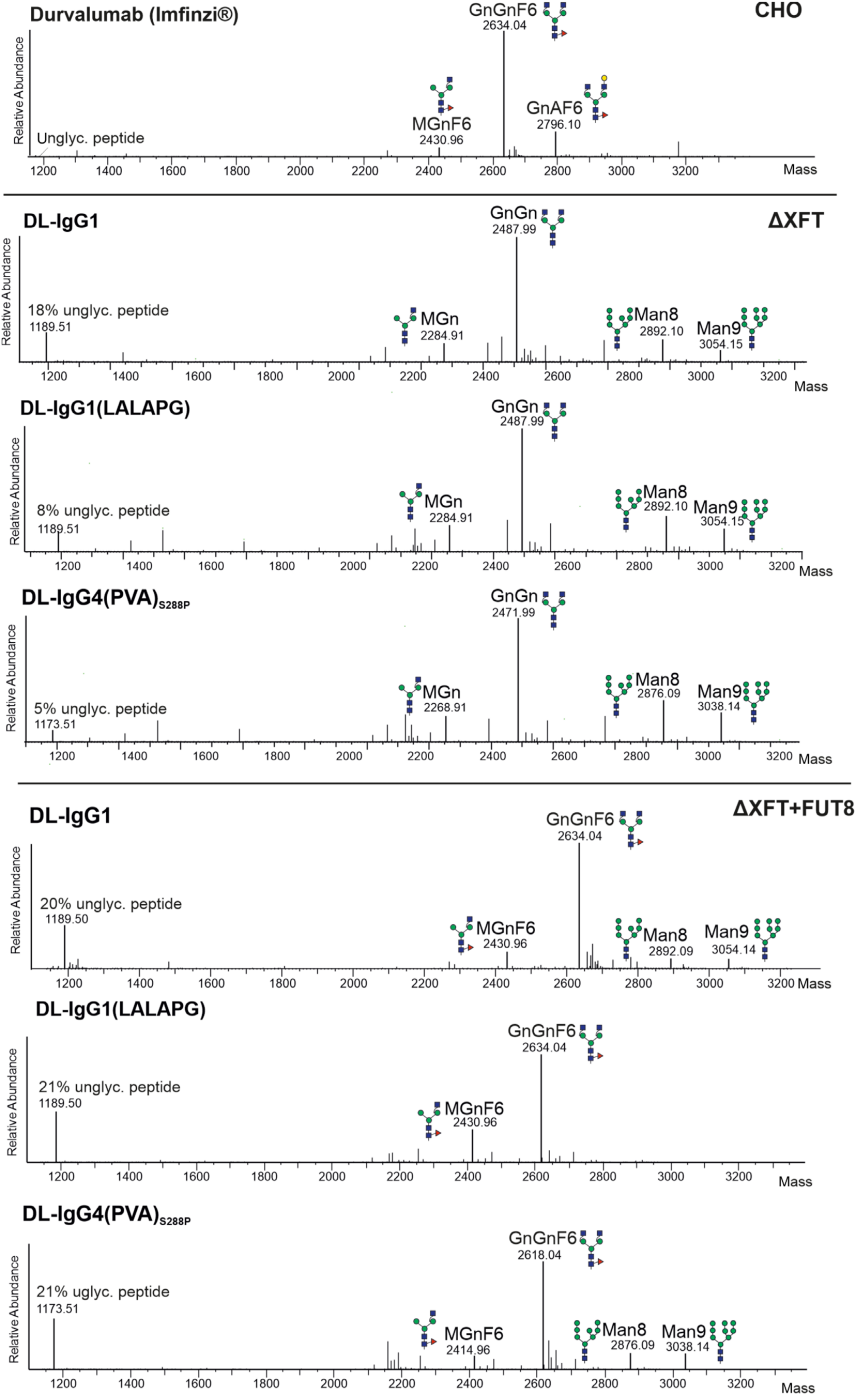
Glycosylation profiles of Durvalumab variants. Glycosylation profiles of Durvalumab (Imfinzi®) and DL variants expressed in *N. benthamiana* ΔXF plants without or with co‐expression of core α1,6‐fucosyltransferase (FUT8) are shown. MS spectra refer to the peptide carrying the N297 N‐glycan: EEQFNSTYR for DL‐IgG4(PVA)_S288P_ and EEQYNSTYR for the other variants. The assigned N‐glycan structures were labelled according to the ProGlycAn nomenclature. A cartoon illustration highlights the main glycan structures detected for each peptide. For details see http://www.functionalglycomics.org/.

DL produced in CHO cells is glycosylated with nearly all glycans core fucosylated (Figure 2). In contrast to mammalian cells that synthesize core fucosylation in α1,6‐linkage, plant *N*‐glycans are modified with α1,3‐linked core fucose residues. Here, we transiently expressed DL variants in *N. benthamiana* ∆XF to produce afucosylated DL and over‐express the human α1,6‐fucosyltransferase (FUT8, Castilho *et al*., 2015) to generate DL glyco‐variants with human‐like core fucosylation. Liquid chromatography–electrospray ionization–mass spectrometry (LC‐ESI‐MS) was used to compare the *N*‐glycosylation profile of DL (Imfinzi^®^) and plant‐derived DL glyco‐variants (Figure 2). DL HC variants expressed in *N. benthamiana* ∆XF are mainly decorated with *N*‐glycans terminated by *N*‐acetylglucosamine residues (GnGn) and lacking core fucose residues while co‐expression of FUT8 resulted in the synthesis of GnGn structures decorated with human‐like core α1,6‐fucose (GnGnF6) and thus resembling DL (Imfinzi^®^). High‐mannose glycans (Man8 and Man9) were also detected in plant‐derived DL variants (up to 25%). Compared to DL (Imfinzi^®^) (0.5%), the percentage of unglycosylated protein is higher for plant‐derived DL (up to 20%) (Figure 2). This underglycosylation is probably due to insufficient activity of the endogenous oligosaccharyltransferase (OST) complex responsible for the *en bloc* transfer of the *N*‐glycan precursor Glc_3_Man_9_GlcNAc_2_ to the consensus sequence for N‐glycosylation of nascent polypeptides. Indeed, we have shown that expression of the single‐subunit OST of *Leishmania major* (LmSTT3D) in plants substantially improves the *N*‐glycosylation efficiency of several recombinant proteins including antibodies (Castilho *et al*., 2018).

### Expression and glycoengineering of PD‐L1

The human programmed cell death 1 ligand (PD‐L1) consists of an ectodomain encompassing an immunoglobulin V‐like domain (IgV), an immunoglobulin C‐like domain (IgC), a transmembrane domain (TM) and a short intracellular tail (Dong *et al*., 2002). Here, we expressed the extracellular domain (ECD) of PD‐L1 with a C‐terminal His‐tag (PD‐L1_His_). Secreted proteins accumulating in the apoplastic fluid (AF) and analysed by SDS‐PAGE showed that PD‐L1_His_ is efficiently secreted. A broad protein band of ~40 kDa was detected in the AF of leaves infiltrated with agrobacteria encoding PD‐L1_His_ (Figure S1A). Additional protein bands also visible in the secretome of leaves infiltrated with an empty vector result from agrobacteria infection. Recombinant PD‐L1_His_ was efficiently purified from AF (Figure S1A) with yields of up to 60 μg/mL of AF (which corresponds to ~40 mg/kg leaf wet weight). The apparent molecular weight of plant‐derived PD‐L1_His_ is higher than its calculated molecular mass (~33 kDa), which is most probably due to protein glycosylation. Indeed, a shift in protein size is observed upon PNGase F digestion providing strong evidence that plant‐derived PD‐L1_His_ is glycosylated (Figure S1A).

The soluble ECD of PD‐L1 has been suggested to form weak homodimers (Chen *et al*., 2010). SDS‐PAGE analysis under non‐reducing conditions revealed that the plant‐derived PD‐L1_His_ is mainly monomeric (Figure S1B).

PD‐L1 carries four potential *N*‐glycosylation sites (N35, N192, N200 and N219) within the ECD. LC‐ESI‐MS analysis of PD‐L1_His_ expressed in *N. benthamiana* ∆XF showed that the protein is mainly decorated with complex neutral glycans (GnGn). Mannosidic (Man5) and hybrid (Man5Gn) glycans were only found for glycosite N192. Overall, the extent of non‐glycosylation is rather high (up to 40%) and glycosite N219 is mostly non‐glycosylated (Figure S1C).

### Binding of Durvalumab variants to PD‐L1

The ability of plant‐derived DL variants to bind to recombinant PD‐L1_His_ was evaluated by immunoblotting and enzyme‐linked immunosorbent assays (ELISA). Western blot results showed that all three DL variants specifically bind PD‐L1_His_ (Figure S2A). Half maximal effective concentration (EC_50_) values in ELISA binding assays confirmed that all plant‐derived DL variants have similar PD‐L1_His_ binding capacities as DL (Imfinzi^®^) (Table 1 and Figure S2B). Overall, these results indicate the integrity of the plant‐produced DL proteins.

**Table 1:** Binding of Durvalumab variants to plant‐derived PD‐L1_His_

Antibody	Glycoform	EC_50_ (ng/mL)	EC_50_ (nm)
DL‐IgG1	GnGn	6.6 ± 0.1	0.04
DL‐IgG1(LALAPG)		6.1 ± 0.1	0.04
DL‐IgG4(PVA)_S288P_		1.6 ± 0.1	0.01
DL‐IgG1	GnGnF6	1.8 ± 0.1	0.01
DL‐IgG1(LALAPG)		9.1 ± 0.1	0.06
DL‐IgG4(PVA)_S288P_		9.5 ± 0.1	0.06
Durvalumab (Imfinzi®)		1.6 ± 0.1	0.01
Nivolumab(C^−^)	na	>2000	>15

Binding of plant‐derived PD‐L1_His_ to Durvalumab (Imfinzi^®^) and to DL variants (DL‐IgG1, DL‐IgG1 (LALAPG) and DL‐IgG4 (PVA)_S288P_) expressed *N. benthamiana* ΔXF plants without (GnGn) or with co‐expressed core α1,6‐fucosyltransferase (GnGnF6) was determined by ELISA (see Figure S1B). EC_50_ (nm) values show similar binding capacity of all DL variants. Nivolumab, (Opdivo^®^, PD‐1 antibody) used as a negative control, does not bind to PD‐L1_His_.

**Figure 3:**
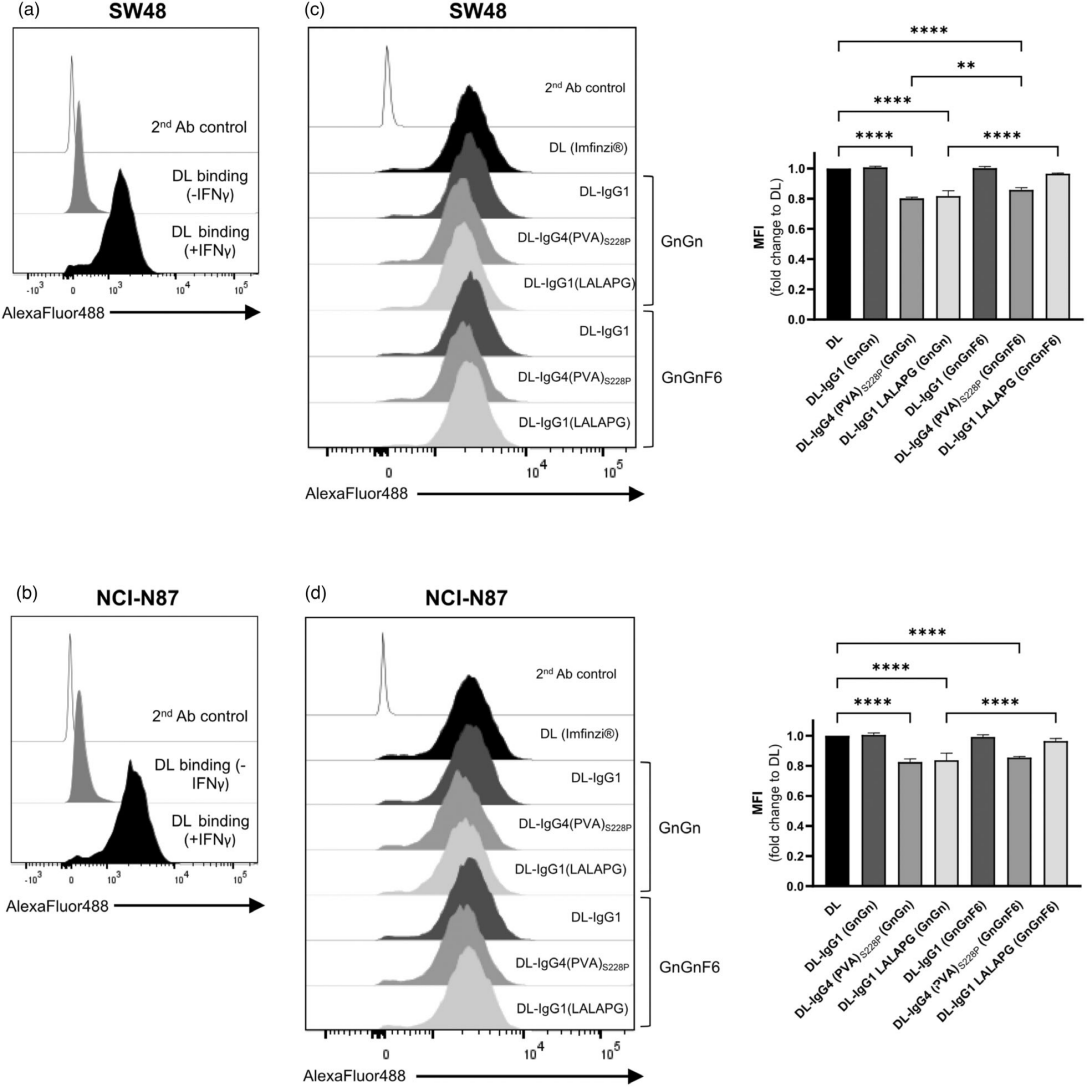
Binding of Durvalumab variants to PD‐L1‐expressing gastrointestinal cancer cells. (a,b) *In vitro* IFNγ stimulation induces the cell surface expression of PD‐L1 and allows the binding of clinical‐grade DL to the NCI‐N87 and SW48 gastrointestinal cancer cell lines. (c,d) The binding of plant‐derived DL variants to cell surface PD‐L1 was assessed via flow cytometry in NCI‐N87 and SW48 cells. The variants DL‐IgG1 (GnGn), DL‐IgG1 (GnGnF6) and DL‐IgG1 (LALAPG) (GnGnF6) showed similar binding as Durvalumab (Imfinzi®), while the variants DL‐IgG4 (PVA)_S288P_ (GnGn), DL‐IgG1 (LALAPG) (GnGn) and DL‐IgG4 (PVA)_S288P_ (GnGnF6) showed a 20% decrease in the binding to cell surface PD‐L1 expressed by cancer cells.

Alterations in glycosylation patterns, particularly increased sialylation, are a hallmark of cancer cells (Bellis *et al*., 2022; Pinho and Reis, 2015; Thomas *et al*., 2021). It has been shown that the steric hindrance of PD‐L1 glycosylation significantly affects antibody recognition, with implications in patient therapeutic stratification (Lee *et al*., 2019).Therefore, we next evaluated the ability of plant‐derived DL variants to bind to cell surface PD‐L1 expressed by two gastrointestinal cancer cells upon *in vitro* interferon gamma (IFNγ) stimulation: the metastatic gastric carcinoma cell line NCI‐N87 and the colorectal carcinoma cell line SW48. Both cell lines have low basal expression of PD‐L1, as demonstrated by flow cytometry (Figure 3a,b). Following *in vitro* IFNγ stimulation, both cell lines start expressing similar PD‐L1 levels at the cell surface in more than 80% of cells. We then assessed the ability of plant‐derived DL variants to bind to PD‐L1 expressing NCI‐N87 and SW48 cells. Both cell lines showed significant binding to all DL variants and clinical‐grade DL (Imfinzi^®^) (Figure 3c,d). DL‐IgG1 (GnGn), DL‐IgG1 (GnGnF6) and DL‐IgG1 (LALAPG) (GnGnF6) showed similar binding to clinical‐grade DL. The variants DL‐IgG4 (PVA)_S288P_ (GnGn), DL‐IgG1 (LALAPG) (GnGn) and DL‐IgG4 (PVA)_S288P_ (GnGnF6) showed 20% decreased binding to both cell lines, when compared to clinical‐grade DL. Overall, the binding of plant‐derived DL variants to cell surface PD‐L1 was not affected by IgG isotype. Fc core fucosylation, however, influenced the binding of some plant‐derived DL variants. In particular, there was a significant increase in the binding of DL‐IgG1 (LALAPG) (GnGnF6) to both cell lines as compared to DL‐IgG1 (LALAPG) (GnGn) (Figure 3c,d).

### Functional evaluation of Durvalumab variants

**Figure 4:**
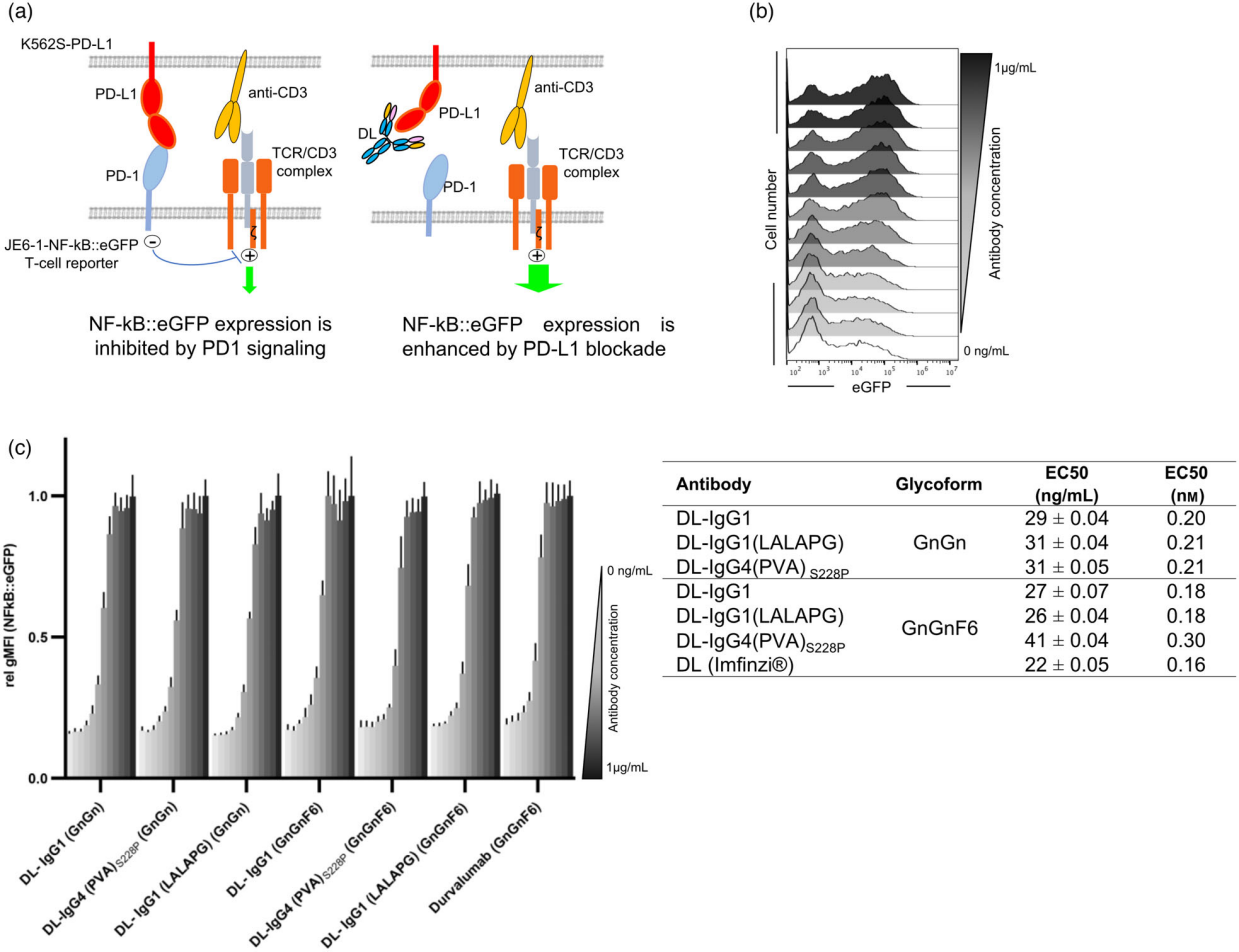
Blocking PD‐1/PD‐L1 interaction by Durvalumab. (a) Simplified schematic representation of the use of a transcriptional PD‐1^+^NF‐κB::eGFP reporter T‐cell line to evaluate the impact of plant‐derived DL on T‐cell activation. Cross‐linking of the TCR‐CD3 complex with CD3‐antibody fragments expressed on K562‐based stimulator cells (K562S‐PD‐L1) results in a strong expression of the eGFP reporter gene. Negative co‐stimulatory signals induced by engagement of PD‐1 with K562S expressing PD‐L1 lead to the inhibition of TCR/CD3 signalling and reduced eGFP expression. Antibodies targeting PD‐L1 block PD‐1 engagement and restore eGFP expression. (b) Example of flow cytometric measurement of eGFP expression in stimulated PD‐1 reporter cells in the absence or presence of Durvalumab. (c) PD‐1 expressing NF‐κB::eGFP reporter cells were stimulated for 24 h with K562S‐PD‐L1 in presence of Durvalumab variants (DL‐IgG1, DL‐IgG1 (LALAPG) and DL‐IgG4 (PVA)_S288P_) and glyco‐variants (GnGn and GnGnF6) at indicated concentrations (1000 to 1 ng/mL). Untreated PD‐1 reporter cells stimulated with K562S‐PD‐L1 used as controls. Data are derived from two independent experiments performed in triplicates (*n* = 6). Data were normalized to the eGFP expression of PD‐1 reporter cells stimulated under conditions where PD‐L1 was fully blocked (1 μg/mL of PD‐L1 antibody). Inhibition curves (Figure S2) and half maximum effective concentrations (EC_50_) were calculated from normalized data using a 4‐parameter logistic function.

We used a previously described PD‐1 reporter T‐cell line (JE6‐1‐NF‐kB::eGFP‐PD1) to evaluate the antagonistic capacity of the plant‐expressed DL variants in a functional assay (De Sousa Linhares *et al*., 2019). The PD‐1 reporter cells were co‐cultured with K562‐based stimulator cells (K562S) expressing a membrane‐bound anti‐CD3 fragment, which engages the TCR‐CD3 complex on the reporter T cells, thereby inducing NF‐kB::eGFP reporter gene expression. K562S‐PD‐L1 co‐expresses PD‐L1, which engages PD‐1 on the reporter cells resulting in a significant inhibition of eGFP expression (Figure 4a). The presence of DL (Imfinzi^®^) blocked PD‐1 inhibition resulting in a dose‐dependent increase in eGFP expression (Figure 4b). Plant‐produced DL variants also efficiently reverted PD‐1 mediated reporter inhibition similarly to the therapeutic antibody and independently of Fc glycosylation (Figure 4c and Figure S3A). Compared to other DL variants, EC_50_ values for fucosylated DL‐IgG4 (PVA)_S288P_ were slightly higher. Similar results were obtained with a different batch of fucosylated DL‐IgG4 (PVA)_S288P_ excluding a batch effect. Interestingly, the EC_50_ values obtained for the fucosylated DL‐IgG4_S228P_ variant lacking the PVA mutation were also higher (EC50 41.4) (Figure S3B).

### Binding of DL variants to human Fcγ receptors

The choice of IgG heavy chain subclass and mutations of Fc ‘hotspots’, particularly at the hinge and upper CH2 domains, strongly affects the binding of antibodies to Fcγ receptors and therefore has utmost pharmacological significance. In addition, Fc *N*‐glycosylation has been shown to affect the affinity of IgG for all FcγR classes (CD64, CD32 and CD16) and to C1q (Hayes *et al*., 2014). We used a flow cytometry‐based assay to test the binding of our plant‐derived DL variants to cells expressing high levels of different human FcγRs—CD16A, the natural V176 high‐affinity variant of CD16A (C16V), CD32A, CD32B and CD64 (Leitner *et al*., 2023). Cells not expressing human FcγR were used as a control.

**Figure 5:**
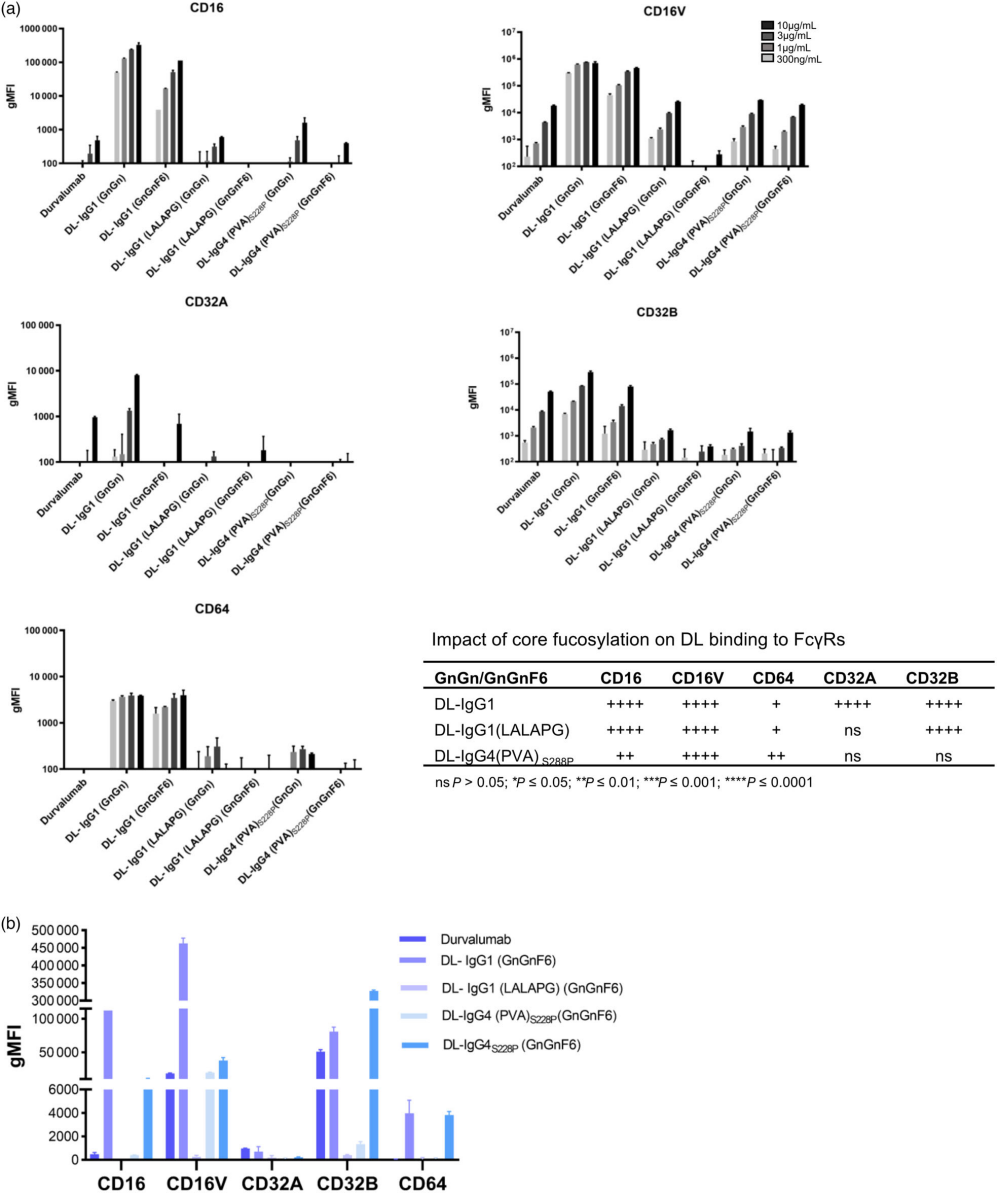
Binding of Durvalumab variants to different FcγRs. (a) Plant‐derived Durvalumab variants (DL‐IgG1, DL‐IgG1 (LALAPG) and DL‐IgG4 (PVA)_S288P_) and glyco‐variants (GnGn and GnGnF6) were compared to DL (Imfinzi®) regarding their ability to bind to cells expressing CD16, CD16V, CD32A, CD32B and CD64 Fcγ receptors. Binding was determined at indicated antibody concentrations (0.3–10 μg/mL) and bound antibodies were detected using an Allophycocyanin‐conjugated AffiniPure F(ab)2 Fragment of Donkey Anti‐Human IgG (H + L specific). Cells not expressing human FcγR served as control to normalize data. Two independent experiments were performed in duplicates. The table summarizes the reduction of binding to FcγR upon core fucosylation. Statistical analysis was performed with multiple comparisons two‐way ANOVA (*n* = 2). (b) Comparison of the binding of DL and plant‐derived core‐fucosylated (GnGnF6) DL variants (used at 10 μg/mL) to different FcγRs.

Overall, DL‐IgG1 showed the highest binding to all FcγRs independently of glycosylation, although core fucosylation reduces the affinity (Figure 5a and Figure S4A). Core fucosylation also significantly decreased the affinity of DL‐IgG1 (LALAPG) to all receptors except for CD32A (Figure 5a and Figure S4B) and the binding of DL‐IgG4 (PVA)_S288P_ particularly to CD16 and CD64 (Figure 5a and Figure S4C).

In order to evaluate the impact of the mutations introduced into DL‐IgG4 (PVA)_S288P_ on FcγR binding, we compared it to DL‐IgG4_S228P_. The results showed that the E233P/F234V/P235A substitutions had no impact on binding to CD32A, while affinity to all other receptors was drastically decreased (up to 30‐fold) (Figure S4D).

Figure 5b compares the FcγR binding of fucosylated DL variants at high concentration (10 μg/mL). Compared to DL (Imfinzi^®^), the binding of fucosylated DL‐IgG4(PVA)_S288P_ and DL‐IgG1(LALAPG) to CD64 was the same (no binding); lower to CD32A and significantly lower to CD32B (40‐fold and 150‐fold, respectively). Notably, the binding of DL‐IgG1 (LALAPG) to CD16V was significantly lower than DL (Imfinzi^®^) and DL‐IgG4 (PVA)_S288P_ (up to 90‐fold).

In summary, fucosylated DL‐IgG1 (LALAPG) showed low affinity to all receptors, and interestingly, the affinity to CD16V and CD32B was significantly lower than its DL (Imfinzi^®^) counterpart which harbours the L234F/L235E/P331S mutations.

### Binding to human FcRn

The circulating half‐life of serum antibodies is roughly 10–21 days, depending on IgG isotype and attributes of the variable region. Fc‐silent DL (Imfinzi^®^) is within this range but a considerably lower half‐life was reported for Avelumab, a wild‐type IgG1 (De Sousa Linhares *et al*., 2019). The Fc contains the binding site for the neonatal Fc receptor (FcRn), a key determinant in extending the plasma half‐life of antibodies by recycling them through transcytosis pathways (Pyzik *et al*., 2019).

Enhanced affinity to FcRn has been achieved through Fc mutagenesis approaches, and the M252Y/S254T/T256E (YTE) triple mutation (Dall'Acqua *et al*., 2002) has consistently shown a twofold to 11‐fold improvement in FcRn binding at pH 6.0.

Surface plasmon resonance (SPR) was used to determine the binding kinetics of human FcRn to DL variants decorated with core α1,6‐fucosylated glycans (GnGnF6). The results (Table 2 and Table S1 and Figure S5) showed that DL (Imfinzi^®^) and DL‐IgG4 (PVA)_S288P_ have K_D_ values higher than the other variants. To determine whether mutations introduced in DL‐IgG4 (PVA)_S288P_ were affecting its binding to FcRn, we also analysed DL‐IgG4_S228P_, which displayed a similar K_D_ value as its PVA counterpart (Table 2 and Table S1 and Figure S5). DL‐IgG1 has a slightly lower K_D_ value and DL‐IgG1(LALAPG) carrying the additional YTE triple mutation exhibited a fourfold increase in affinity to hFcRn compared to therapeutic DL (Imfinzi^®^) (Table 2 and Table S1 and Figure S5).

**Table 2:** Binding of Durvalumab to hFcRn

Antibody	K_D_ (nm)	Rmax (RU)
DL‐IgG1	92 ± 3	22.2 ± 0.6
DL‐IgG1(LALAPG)	45 ± 2	23.7 ± 0.2
DL‐IgG4(PVA)_S228P_	129 ± 9	25.5 ± 0.6
DL‐IgG4_S228P_	162 ± 5	18.6 ± 0.4
Durvalumab (Imfinzi®)	168 ± 6	27.6 ± 0.1

SPR was used to compare the binding of the human neonatal receptor to Durvalumab (Imfinzi®) and DL variants (DL‐IgG1, DL‐IgG1 (LALAPG) and DL‐IgG4 (PVA)_S288P_ expressed in *N. benthamiana* ΔXF plants together with core α1,6‐fucosyltransferase (GnGnF6). Equilibrium dissociation constants (K_D_) in nm and the binding response (expressed as RUs, Response Units) are shown as the mean and standard deviation (SD) of three separate runs (mean ± SD). For more information see also Figure S4 and Table S1.

## Discussion

The PD‐1/PD‐L1 immune regulatory axis has a central role in the suppression of anti‐tumour immunity (Baumeister *et al*., 2016). In recent years, the therapeutic potential of PD‐1/PD‐L1 blockade has been demonstrated in multiple human clinical trials and achieved remarkable clinical success in multiple solid cancers (Li *et al*., 2018).

In cancer therapy, antibodies of the IgG1 subclass favour tumour destruction through the recruitment of immune effectors. The role of the Fc‐mediated effector functions in cancer therapy using PD‐L1 blocking antibodies has been discussed controversially (Cohen Saban *et al*., 2023; Knorr and Ravetch, 2019; Leitner *et al*., 2021; Yu *et al*., 2020). While current human PD‐1 therapeutics are based on IgG4 to minimize antibody‐dependent cell lysis, with the exception of Avelumab, PD‐L1 blockers are IgG1 mAbs with reduced Fc‐effector function via amino acid substitutions on the FcγR‐binding domain or ‘null’ glycosylation.

The biological activity of mAbs is actively modulated by their glycosylation profile (Mastrangeli *et al*., 2019). Fc core fucosylation is implicated in FcγRIIIa binding and negatively affects ADCC activity; terminal galactose was reported to be involved in FcγRIIIa binding but not impacting ADCC activity, while the function of terminal sialylation is still being discussed (Mastrangeli *et al*., 2019; Park *et al*., 2020).

Improving efficacy and prolonging half‐life is an important strategy to make checkpoint inhibitors more accessible and affordable. Mammalian cells are traditionally used for mAb production. However, in recent years, plants have carved their way as suitable alternative expression platforms. Plant expression systems have several advantages over conventional expression systems. The cultivation of plants under controlled conditions ensures the production of high‐quality protein in accordance with GMP (Good Manufacturing Practice) standards at lower costs (Buyel and Fischer, 2012; Diamos *et al*., 2019; Ecker *et al*., 2015; Ma *et al*., 2017; Ridgley *et al*., 2023). Moreover, the rapid growth, low cost of nutrient components and the avoidance of risks from contaminations with viruses and prions of animal origin are other arguments in favour of plant‐derived therapeutics. Another important benefit of plant‐based systems is that in comparison with mammalian‐derived proteins which have a highly heterogeneous glycosylation, plant‐derived therapeutics normally carry well‐defined, homogeneous and consistent glycosylation patterns. Transient expression in *N. benthamiana* is particularly well suited for the generation of human antibodies at high yields and with tailored glycosylation profiles (Castilho *et al*., 2011, 2015; Loos and Steinkellner, 2012; Montero‐Morales and Steinkellner, 2018).

*Nicotiana benthamiana* plants lacking endogenous β1,2‐xylose and core α1,3‐fucose residues (ΔXF, (Strasser *et al*., 2008) have been used to produce a variety of antibodies with enhanced immune receptor binding and greater potency compared to commercial antibodies, including the well‐described ZMAPP™, an antibody cocktail developed for the treatment of Ebola virus infection (Zeitlin *et al*., 2011). In contrast, blocking antibodies with engineered Fcs designed to eliminate effector functions and reduce dose/administration frequency represent hold promise as replacements of current PD‐1 and PD‐L1 therapeutic antibodies.

So far PD‐L1 and PD‐1 antibodies produced in *N. benthamiana* plants were either decorated with plant‐specific glycan epitopes (core α1,3‐fucose and xylose), afucosylated or carrying high‐mannose glycans (Phakham *et al*., 2021; Phetphoung *et al*., 2022; Rattanapisit *et al*., 2019; Ridgley *et al*., 2023). The major drawbacks are that (i) mannosylation of therapeutic IgG antibodies increases their serum clearance by mannose receptors (Goetze *et al*., 2011); (ii) classical plant CCD epitopes can lead to host antibody reactivity (Nkurunungi *et al*., 2021); and (iii) lack of core fucose can significantly enhance antibody ADCC against PD‐L1‐positive non‐tumour cells (Yamane‐Ohnuki and Satoh, 2009). Here, we have used *N. benthamiana* ΔXF plants to produce the PD‐L1 therapeutic DL as wild‐type IgG1 and IgG4_S228P_ subclasses. In addition, mutations were introduced to engineer Fc‐null or effector‐*silenced DL* antibody variants. The triple E233P/F234V/P235A (PVA) amino acid substitution present on IgG2 antibodies *was introduced to further reduce the* affinity of *IgG4*_*S228P*_ to FcγRI that naturally has reduced ADCC and ‘null’ CDC (DL‐IgG4 (PVA)_S288P_). The ‘effector‐silent’ DL‐IgG1 (LALAPG) carries additional amino acid substitutions in the Fc region (YTE) that are widely used to prolong half‐life and thus reduce dose and administration frequency (Gautam *et al*., 2018).

DL variants expressed in *N. benthamiana* ΔXF show high integrity with no significant degradation or aggregation. Importantly, DL could be purified with yields up to 1 g/kg of fresh leaves, without the need for optimizing expression. Such recovery rates are significantly higher compared to previous reports on antibody accumulation levels (before purification) of 0.3 g/kg (Phakham *et al*., 2021) and 0.7 g/kg upon co‐expression with a P19 silencing suppressor to optimize expression (Ridgley *et al*., 2023).

Apart from afucosylated DLs, we produced glyco‐variants that truly mimic the glycosylation profile of therapeutic DL. Co‐expression of human α1,6‐fucosyltransferase (FUT8) was an efficient approach to produce homogenously fucosylated DL variants where the α1,3‐linked core fucose typical of plant glycans was substituted by a non‐immunogenic epitope.

All plant‐derived DL variants were able to bind to soluble PD‐L1, produced in *N. benthamiana* ΔXF plants at high yields (40 mg/kg of leaf material). PD‐L1 is aberrantly overexpressed in malignancies of several origins (Dong *et al*., 2002) and possible alterations in the glycosylation pattern of PD‐L1 might be related to the acquisition of molecular resistance to targeted therapeutic antibodies (Benicky *et al*., 2021). One of the most common glycan alterations in malignant cells is hypersialylation (Munkley, 2022). Recent studies showed that increased α2,6‐sialylation of cell surface antigens leads to a marked resistance to therapeutic antibodies in gastrointestinal cancer (Duarte *et al*., 2021; Rodrigues *et al*., 2021; Yen *et al*., 2015). Glyco‐profiling of PD‐L1 expressed in MDA‐MB‐231 breast cancer cells showed that its complex glycans are fully sialylated (Benicky *et al*., 2021).

The pattern of expression and potential of PD‐L1 as an immunohistochemical biomarker has been extensively studied in gastrointestinal neoplasms (Mastracci *et al*., 2022) but a comprehensive glycan analysis has not yet been reported. Here, we show that plant‐derived DL variants are able to bind PD‐L1 expressed in gastric and colorectal cancer cells. Interestingly, compared to DL (Imfinzi^®^) and DL‐IgG1, the binding of DL‐IgG1 (LALAPG) and DL‐IgG4 (PVA)_S288P_ to PD‐L1‐positive gastrointestinal cancer cells was less effective. These differences were not observed in the binding to recombinant soluble PD‐L1 and to PD‐L1 expressed in the human erythroleukaemia K562 cell line. Recent studies have suggested that both variable and constant domains play a role in antibody–antigen recognition (Janda *et al*., 2016; Lua *et al*., 2018). Also, the hinge region allows a high flexibility between the Fab and Fc fragment in order to enhance the accessibility of the Fab fragment for antigen binding (Saphire *et al*., 2002). At the moment, the reasons underlying this unexpected observation are not clear. However, it is known that even minor structural variations (including Fc glycosylation) may affect antibody conformation, which may, in turn, impact antigen binding (Scallon *et al*., 2007; Strasser *et al*., 2009). We cannot exclude that the mutations introduced in IgG1 and IgG4 _S288P_ impact their binding to PD‐L1 on the cell surface. To note, DL (Imfinzi^®^) also carries mutations in the hinge region (L234F/L235E/P331S). Also, the potential steric hindrance of extensive PD‐L1 glycosylation in gastrointestinal cancer cells might affect its recognition by these two variants.

Of note, the cell‐specific glycosylation profile of PD‐L1 can also determine the binding affinity of PD‐L1‐targeting mAbs. Site‐specific glycoproteomic profiling of PD‐L1 in cancer cells will certainly shed light on the molecular role played by receptor site‐specific glycosylation in the differential binding to DL variants and support the design of novel glycoengineered DL variants capable of circumventing glycan‐mediated binding inhibition.

Overall, plant‐derived DL variants showed similar ability to block the PD‐L1/PD‐1 interaction in a dose‐dependent manner, comparable to therapeutic DL and independent from glycosylation and Fc mutations. These structural features, however, had a significant impact on the affinity to Fc receptors. Our results corroborated previous findings and demonstrated that apart from amino acid mutations in the Fc region, glycoengineering can also be used to finely tune the binding affinity to different Fcγ receptors. We found that DL‐IgG1 binds better to all FcγRs, and although core fucosylation drastically reduces its binding affinities, it is not sufficient to generate a Fc‐effector‐*silenced* IgG.

IgG4 antibodies have naturally reduced affinities for most FcγRs and C1q (Crescioli *et al*., 2016; Jiang *et al*., 2011) but still retain high affinity to FcγRI and binding to FcγRIIb, which can result in macrophage‐mediated phagocytosis of PD‐1‐positive T cells (Arlauckas *et al*., 2017) and decreased anti‐tumour activities. DL‐IgG4_S228P_ binds to several Fcγ receptors including CD64. For anti‐PD‐1 antibodies, it has been shown that cross‐linking between PD‐1 and CD64 could change the function of the antibody from blocking to activating (Zhang *et al*., 2018). Here, we show that mutations introduced in DL‐IgG4 (PVA)_S288P_ reduced the binding to CD16, CD64 and CD32B when compared to DL‐IgG4_S228P._ Interestingly, DL‐IgG4(PVA)_S288P_ carries only three out of the six amino acid substitutions previously reported (Zhang *et al*., 2018), showing that these mutations within the hinge region are sufficient to modulate FcγR affinity.

From all DL variants, core‐fucosylated DL‐IgG4 (PVA)_S288P_ and DL‐IgG1 (LALAPG) showed consistently low affinity to all FcγRs. Therapeutic DL (Imfinzi^®^) contains a triple mutation (L234F/L235E/P331S) that causes a profound decrease in its binding to CD64, CD32A, CD16V and C1q (Oganesyan *et al*., 2008). The L234A/L235A/P331G mutations used in this investigation to produce DL‐IgG1 (LALAPG) seem to be significantly effective to further reduce the affinity of DL to CD16V and to CD32B. This could be advantageous, since targeted blockade or genetic depletion of the inhibitory FcγRIIB receptor have been used to overcome therapeutic resistance and boost activity of antibodies in cancer immunotherapy (Cohen Saban *et al*., 2023; Teige *et al*., 2019). Furthermore, introduction of the M252Y/S254T/T256E (YTE) mutations significantly enhanced binding of DL‐IgG1 (LALAPG) to the FcRn at pH 6 without impacting the release at pH 7. Higher affinity for the FcRn is a common indicator of a potentially enhanced half‐life of mAbs.

A recent study generated a production cost model showing that the expression and purification recovery levels using plant platforms were as competitive as mammalian cell‐based platforms and therefore suitable to deliver accessible and more affordable diagnostic and therapeutic proteins (Ridgley *et al*., 2023). Our research consolidates the scientific, clinical and economic potential of plant‐based expression platforms and dampens initial concerns relating to low yields and differences in glycosylation. Importantly, we show that plants can deliver biosimilars to therapeutic DL (Imfinzi^®^) with (i) comparable biological effector functions, (ii) reduced interaction with FcγR and (iii) enhanced binding to FcRn which could result in optimal FcγR‐mediated effector functions and a longer half‐life *in vivo*.

## Material and methods

### Construction of Durvalumab HC variants

The cDNA sequences of wild‐type human IgG1 and IgG4_S228P_ constant heavy chains (CH1‐CH3 domains) lacking the variable regions (VH) and codon optimized for *N. benthamiana* were first cloned into the magnICON^®^ tobacco mosaic virus‐based (TMVα: pICHα26211, (Klimyuk *et al*., 2014)) vectors that include the signal peptide of barley α‐amylase to target proteins to the secretory pathway and two *Bsa*I restriction sites.

Heavy chain variants to generate a Fc‐silent IgG1 (mutations: L234A/L235A/P331G and M252Y/S254T/T256E) and a Fc‐silent IgG4 (mutations: E233P/F234V/P235A with additional S228P/Y219C/G220C substitutions) were obtained by *overlap extension PCR* using primers to introduce mutations at the hinge region and CH2 domain. Silent IgG4 (PVA)_S288P_ and IgG1 (LALAPG) HCs lacking the variable regions were cloned into TMVα vectors.

Similarly, the constant region of the human kappa light chain (LC) lacking the variable region was codon optimized for *N. benthamiana* and cloned into the magnICON^®^ potato *virus* X‐based (PVX α: pICHα31150 vector, Klimyuk *et al*., 2014) carrying the signal peptide of barley α‐amylase and two *Bsa*I restriction sites.

The amino acid sequence for both the light and heavy chains of Durvalumab (DL) can be found in the international ImMunoGeneTics information database (IMGT/mAb‐DB ID 528). Codon‐optimized sequences for the variable regions (VL: 1‐107aa and VH: 1‐121aa) were introduced upstream of HC and LC regions using the *Bsa*I restriction sites.

### Cloning of PD‐L1

The DNA sequence coding for the extracellular domain (aa 18–283, Q9NZQ7) of human programmed cell death 1 ligand (PD‐L1) with a C‐terminal polyhistidine tag was codon optimized for *N. benthamiana* and cloned into viral‐based magnICON^®^ TMVα vectors (PD‐L1_His_).

### Transient expression and protein extraction

Recombinant proteins were expressed in *N. benthamiana* glycosylation mutant plants (ΔXF (Strasser *et al*., 2008)) via agroinfiltration. Infiltrated leaves harvested 4 days postinfiltration (dpi) were used for protein extraction. For detailed information see Appendix S1.

### PD‐L1_His_ purification

For purification of His‐tagged PD‐L1, a 0.7 × 5 cm Econo‐column (Bio‐Rad) was packed with 1 mL of Ni‐NTA His•Bind^®^ resin (Sigma) and equilibrated with 10 column volumes of 20 mm Na2HPO4, 500 mm NaCl, pH 7.4. The apoplastic fluid was loaded to the column at 1 mL/min and the column was washed with 10 volumes of 20 mm Na2HPO4; 500 mm NaCl, 30 mm imidazole, pH7.4. Bound proteins were eluted with 1 mL of 20 mm Na_2_HPO_4_, 500 mm NaCl and 500 mm imidazole (pH 7.4). Fractions containing PD‐L1_His_ were pooled and dialysed overnight against PBS, pH 7.4 using SnakeSkin dialysis tubing (Thermo Fisher Scientific) with a 10 kDa molecular mass cut‐off.

### Antibody purification

DL variants were purified from total soluble protein extracts using an ÄKTA pure protein purification system (Cytiva) and a 5 mL HiTrap Protein A HP (17040301 Cytiva) column. The column was equilibrated with 5 column volumes of 20 mm Tris/HCl, 150 mm NaCl, pH 7.4 (flow rate 4 mL/min) and the sample was loaded at a flow rate of 3 mL/min. After washing the column with 12 column volumes of 20 mm Tris/HCl, 150 mm NaCl, pH 7.4 (flow rate 4 mL/min), proteins were eluted with 5 column volumes of 0.1 M glycine/HCl, pH 3.5 (flow rate 2.5 mL/min), in 1 mL fractions. Pooled eluates were neutralized with 1 M Tris/HCl, pH 8.0 and dialysed against PBS, pH 7.4 as outlined above.

### Size exclusion chromatography

Purified DL samples were concentrated with Amicon Ultra‐0.5 centrifugal filters, MWCO 10 000 kDa (Merck Millipore). To isolate the fully assembled antibody fractions, size exclusion chromatography (SEC) using a HiLoad Superdex 200/10/300 GL column was performed. The column was equilibrated with PBS (pH 7.4). Eluted samples were collected and concentrated with Amicon centrifugal filters. Concentrations were determined spectrophotometrically at a wavelength of 280 nm (NanoDrop™ 2000, Thermo Scientific) (using the extinction coefficients 1.585, 1.606 and 1.572 M^−1^/cm for DL‐IgG1, DL‐IgG1 (LALAPG) and DL‐IgG4 (PVA)_S288P_, respectively).

### Protein analysis

Apoplastic and purified recombinant proteins were fractionated by 10% SDS‐PAGE under reducing and non‐reducing conditions and either stained with Coomassie brilliant blue (CBB, G‐250) or analysed by immunoblotting.

DL variants were analysed by Western blots using both anti‐human gamma chain HRP (1 : 5000, Sigma‐Aldrich, A8775) and anti‐human kappa chain HRP (1 : 5000, Sigma‐Aldrich, A7164). Detection was performed with Clarity™ Western enhanced chemiluminescence reagents (Bio‐Rad), and images were captured with a Fusion Solo S image system (Vilber Lourmat).

### Glycoengineering

DL HC variants were produced in *N. benthamiana* as two glycoforms. Expression of recombinant proteins in *N. benthamiana* ΔXF leads to the generation of afucosylated variants, while co‐expression of the human‐like core α1,6 fucosyltransferase (FUT8) enables the synthesis of core α1,6 fucosylated proteins (Castilho *et al*., 2015).

PD‐L1^His^ was produced in *N. benthamiana* ΔXF as an afucosylated protein.

### Glycan analysis

PD‐L1 and DL were digested in solution with trypsin and subjected to LC‐ESI‐MS analysis (Grunwald‐Gruber *et al*., 2017). Digested peptides were separated using a nanoEase C18 column. Detection was performed with a QTOF MS (maXis 4G, Bruker) equipped with the standard ESI source in positive ion, DDA mode. The possible glycopeptides were identified as sets of peaks consisting of the peptide moiety and the attached N‐glycan varying in the number of HexNAc, hexose, deoxyhexose and pentose residues. Manual glycopeptide searches were performed using FreeStyle 1.8 (Thermo Scientific); deconvolution was done using the extract function.

### Enzyme‐linked immunosorbent assays (ELISA)

The binding properties of plant‐derived DL variants (and controls) to recombinant PD‐L1_His_ were determined by ELISA in three technical replicates (see Appendix S1).

### Binding of plant‐derived DL variants to gastrointestinal cancer cells

For functional assays, NCI‐N87 gastric cancer and SW48 colorectal cancer cells were seeded in 6‐well culture plates, in complete growth medium, and supplemented with IFNγ (40 ng/mL) to induce the cell surface expression of PD‐L1. Following 48 h of IFNγ stimulation, cells were harvested and their reactivity to clinical‐grade DL and plant‐derived DL variants was evaluated by flow cytometry. The detailed cell line description and flow cytometry assay can be found in Appendix S1.

### Cell reporter assays

Details of the culture of the reporter cell lines and flow cytometry can be found in Appendix S1.

JE6‐1‐NF‐kB::eGFP‐PD‐1 reporter cells (5 × 10^4^) were co‐cultured with K562S‐PD‐L1 in round‐bottom 96‐well plates in the presence or absence of DL variants used at 1000, 500, 250, 125, 62.5, 31.25, 15.63, 7.81, 3.91, 1.95 and 0.98 ng/mL (twofold dilution steps). Following 24 h of co‐culture, plates were measured by flow cytometry. The geometric mean of the fluorescence intensity (gMFI) of viable reporter cells was used for further analysis. K562S cells were excluded based on their expression of red fluorescent protein (RFP). Data were normalized to the eGFP expression of PD‐1 reporter cells stimulated under conditions where PD‐L1 was fully blocked (1 μg/mL of PD‐L1 antibody).

### Binding to FcγRs

To evaluate the binding of DL variants to human FcγRs, we used a set of BW5147 lines stably expressing high levels of human FcγR—CD16A (FcγRIIIA), the natural V176 high‐affinity variant of CD16A (FcγRIIIA 176 V), CD32A (FcγRIIA), CD32B (FcγRIIB) and CD64 (FcγRI) (Leitner *et al*., 2023). BW5147 cells not expressing human FcγR were used as a control. FcγR‐expressing and control cells were incubated with different concentrations of DL variants (0.3; 1; 3 and 10 μg/mL) for 30 min at 4 °C. Following a washing step bound, antibodies were detected using Allophycocyanin‐conjugated AffiniPure F(ab)2 Fragment of Donkey Anti‐Human IgG (H + L specific). Cells were subjected to another washing step and measured by flow cytometry.

### Binding to FcRn

Binding of DL variants to hFcRn was determined by surface plasmon resonance (SPR) in three replicates, using the Biacore T200 system (GE Healthcare) at 25 °C. A Biacore CM5 Sensor Chip (*Cytiva*) was directly coated with 2.5 μg/mL of hFcRn (R&D Systems, 8639‐FC‐050, P55899) using an amine coupling kit (*Cytiva*, BR‐1000‐50) to approximately 80 response units (RU). PBS (pH 6) supplemented with 0.05% Tween‐20 was used as running buffer. Plant‐derived DL variants were injected at 6.87–440 nm for 60 s and allowed to dissociate for 60 s. DL (Imfinzi^®^) was used as control. The chip was regenerated in PBS (pH 7.4). The binding kinetics, *k*_on_ (1/Ms), *k*_off_ (1/s) and K_D_ (nm) were calculated from global fittings using a 1 : 1 binding model (BIAevaluation software). In the case of a very fast on‐rate and a very fast off‐rate, the K_D_ value was calculated from steady state affinity measurements.

### Statistics

EC_50_ values were estimated by non‐linear regression based on a four‐parameter logistic curve (4PL) model with GraphPad Prism (version 9).

Statistical analysis was performed with multiple comparisons one‐ and two‐way ANOVA.

## Conflict of interest

The authors declare that the research was conducted in the absence of any commercial or financial relationships that could be construed as a potential conflict of interest.

## Funding

This work was supported by grants from the Austrian Science Fund (FWF‐P35292‐B to A.C.; FWF‐P32411‐B to P.S.) and by the Portuguese Foundation for Science and Technology (FCT): PTDC/MEC‐ONC/0491/2021 grant to C.A.R. and 2022.04138.PTDC to H.O.D. H.O.D. was supported by a Junior Researcher contract (2022.00943.CEECIND) awarded by FCT.

## Author contributions

All authors have made a substantial intellectual contribution to the work. SI, SG, PC, JG and HOD conducted experimental investigation. AC, PS, LM, JG and CAR conceptualized and designed the experiments. VK propagated the plants and did agroinfiltration. JL generated essential cell lines. All authors were involved in data analysis and writing of the manuscript. They all approved it for publication.

## Supporting information


**Figure S1** Binding of Durvalumab variants to PD‐L1_His_.
**Figure S2** Expression, purification and glycosylation of plant‐derived PD‐L1_His_.
**Figure S3** Blocking PD‐1/PD‐L1 interaction by Durvalumab.
**Figure S4** Binding of Durvalumab variants to Fcγ receptors.
**Figure S5** SPR sensorgrams for the binding of Durvalumab variants to hFcRn.
**Table S1** Kinetic parameters of the binding of Durvalumab variants to hFcRn.

**Appendix S1** Transient expression‐agroinfiltration.

## Data Availability

Data sharing not applicable to this article as no datasets were generated or analysed during the current study.
