# Supplementary material for: Plant‐derived Durvalumab variants show efficient PD‐1/PD‐L1 blockade and therapeutically favourable FcR binding
Source: Plant Biotechnol J. 2023 Dec 4;22(5):1224–37. doi: 10.1111/pbi.14260 (PMC11022803; doi:10.1111/pbi.14260)
Supplement: Supplementary file 1 — Figure S1 Binding of Durvalumab variants to PD‐L1His. Figure S2 Expression, purification and glycosylation of plant‐derived PD‐L1His. Figure S3 Blocking PD‐1/PD‐L1 interaction by Durvalumab. Figure S4 Binding of Durvalumab variants to Fcγ receptors. Figure S5 SPR sensorgrams for the binding of Durvalumab variants to hFcRn. Table S1 Kinetic parameters of the binding of Durvalumab variants to hFcRn. [file PBI-22-1224-s001.zip › Supplementary Figures.pdf]

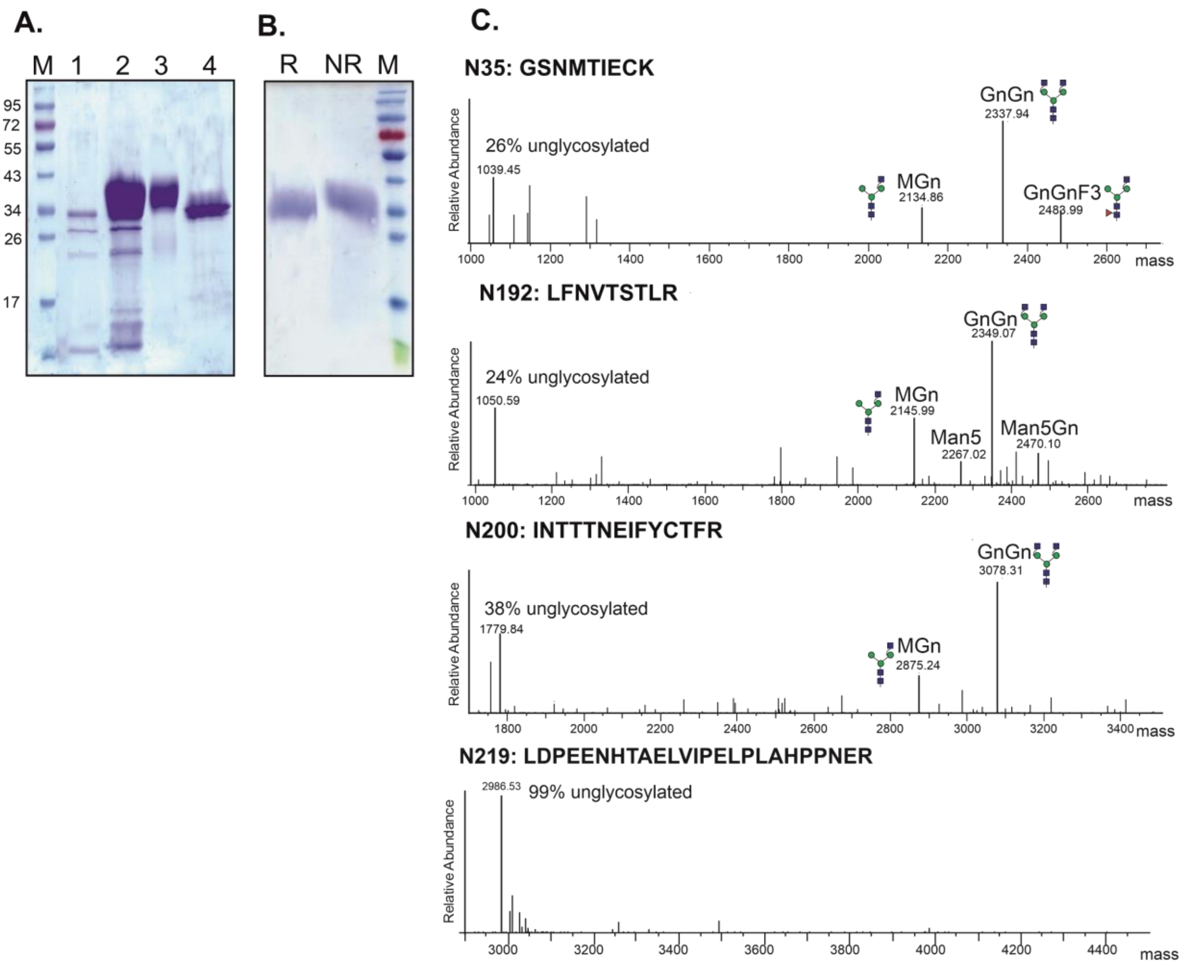

Figure S1: Expression, purification and glycosylation of plant-derived PD-L1$_{His}$

(A) SDS-PAGE analysis (R: reduced; NR: non-reduced) and Coomassie brilliant blue staining of proteins isolated from the apoplastic fluid (AF) of leaves infiltrated with agrobacteria carrying (1) an “empty” TMVα plasmid and (2) TMVαPD-L1$_{His}$. A protein band corresponding to PD-L1$_{His}$ is detected at ~40 kDa. Additional protein bands represent proteins derived from agrobacterial infection. PD-L1$_{His}$ isolated by affinity chromatography was analysed before (3) and after (4) enzymatic deglycosylation with PNGase F. The apparent molecular mass of marker proteins are shown in kilo Dalton (kDa). (**B**) SDS-PAGE run under reducing (R) and non-reducing (NR) conditions shows that plant-derived PD-L1$_{His}$ is monomeric. (**C**) LC-ESI-MS glycosylation profile of PD-L1$_{His}$ expressed in *N. benthamiana* ΔXF plants. The relative abundance of the respective non-glycosylated peptide is shown. The assigned N-glycans are labelled according to the ProGlycAn nomenclature. A cartoon illustration highlights the main glycan structures detected for each peptide. For details see http://www.functionalglycomics.org/.

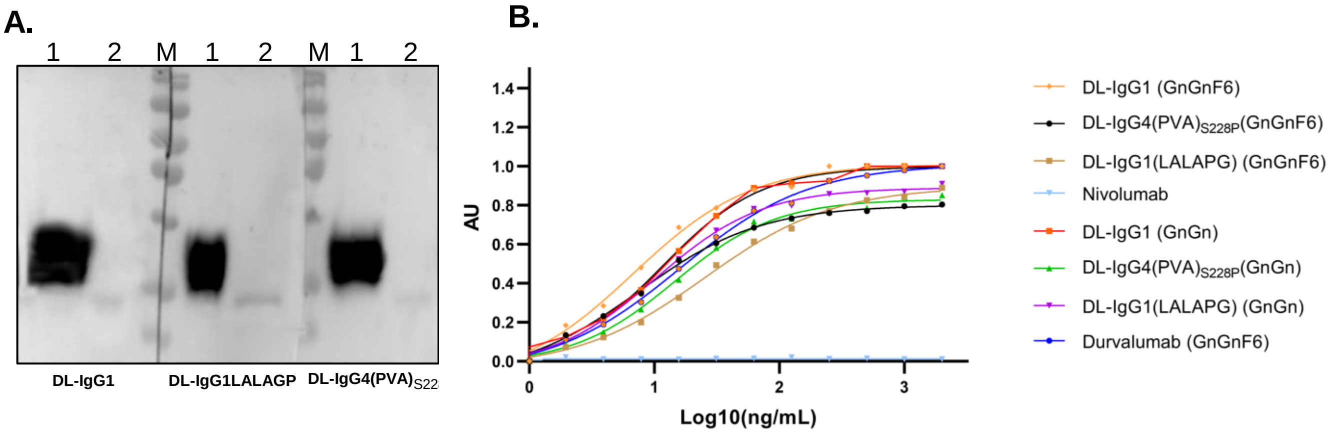

Figure S2: Binding of Durvalumab variants to PD-L1$_{His}$

(A) Immunoblotting of PD-L1$_{His}$ (1) and an unrelated His-tagged protein (2), probed with DL variants and detected with anti-gamma-HRP antibodies shows that all three DL variants specifically bind PD-L1$_{His}$. **(B)** Binding of plant-produced fucosylated and afucosylated DL HC-variants to recombinant PD-L1$_{His}$. Dilutions of plant-produced DL were incubated on plates coated with PD-L1$_{His}$ and detected with anti-human IgG-HRP. Durvalumab (Imfinzi®) and Nivolumab (Opdivo®, PD1 antibody) were used as positive and negative controls, respectively. Data represent the mean values of duplicates.

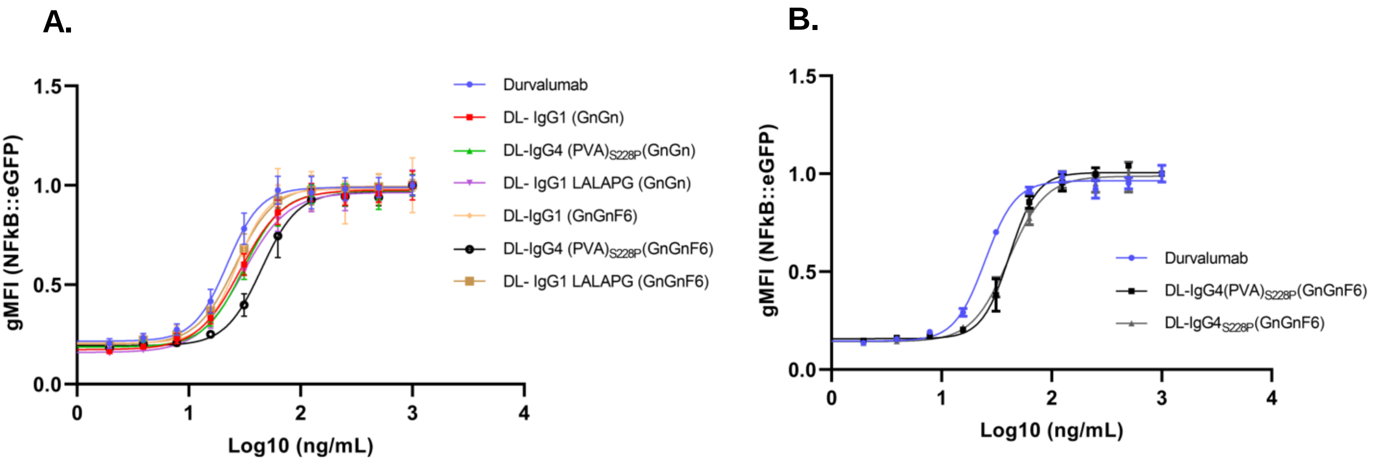

Figure S3: Blocking PD-1/PD-L1 interaction by Durvalumab

Inhibition curves used to determine functional half maximum effective concentrations (EC_{50}). DL variants were evaluated at different concentrations (ranging from 1 to 1000 ng/mL) using PD-1^{+}NF-κB::eGFP T-cell reporters cocultured with T cell stimulator cells expressing PD-L1. **(A)** Comparison of afucosylated (GnGn) and fucosylated (GnGnF6) DL-IgG1, DL-IgG1(LALAPG) and DL-IgG4(PVA)$_{S288P}$ with Durvalumab (Imfinzi®). **(B)** Comparison of fucosylated (GnGnF6) DL-IgG4(PVA)$_{S288P}$ and DL-IgG4$_{S228P}$ with Durvalumab (Imfinzi®). gMFI, geometric mean of fluorescence intensity.

Figure S4

A. DL-IgG1

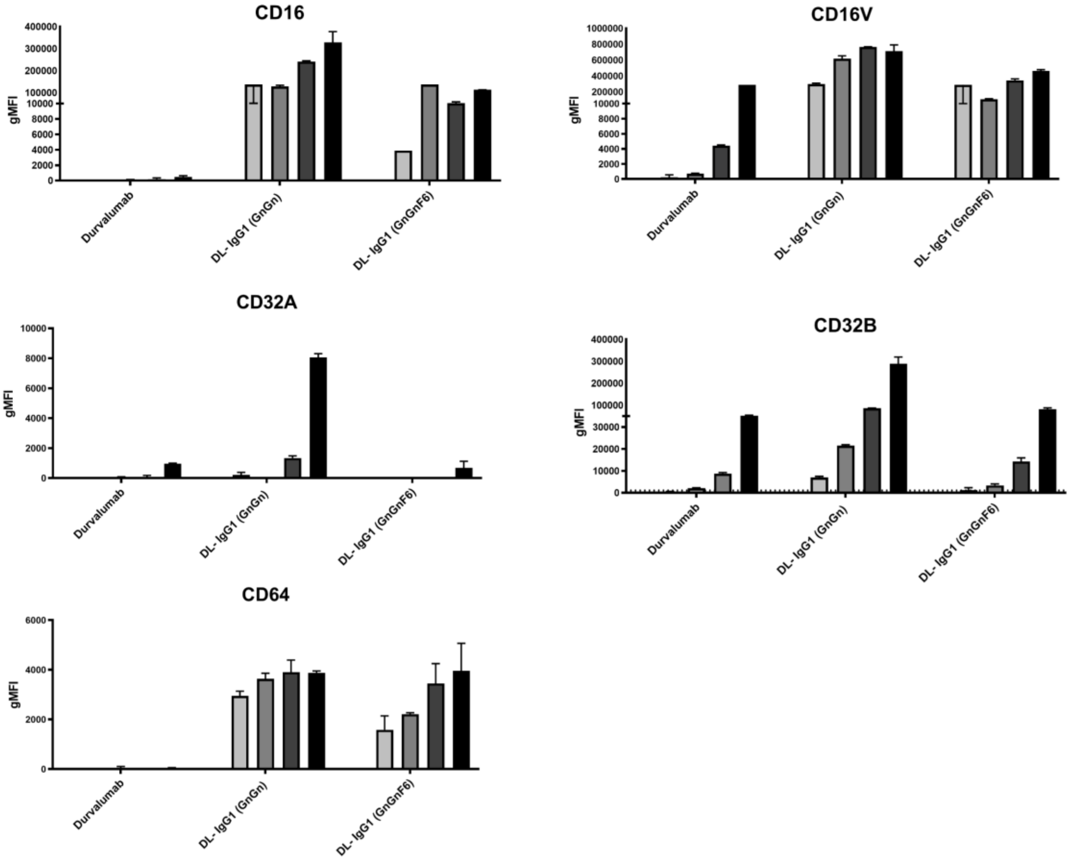

B. DL-IgG1(LALAPG)

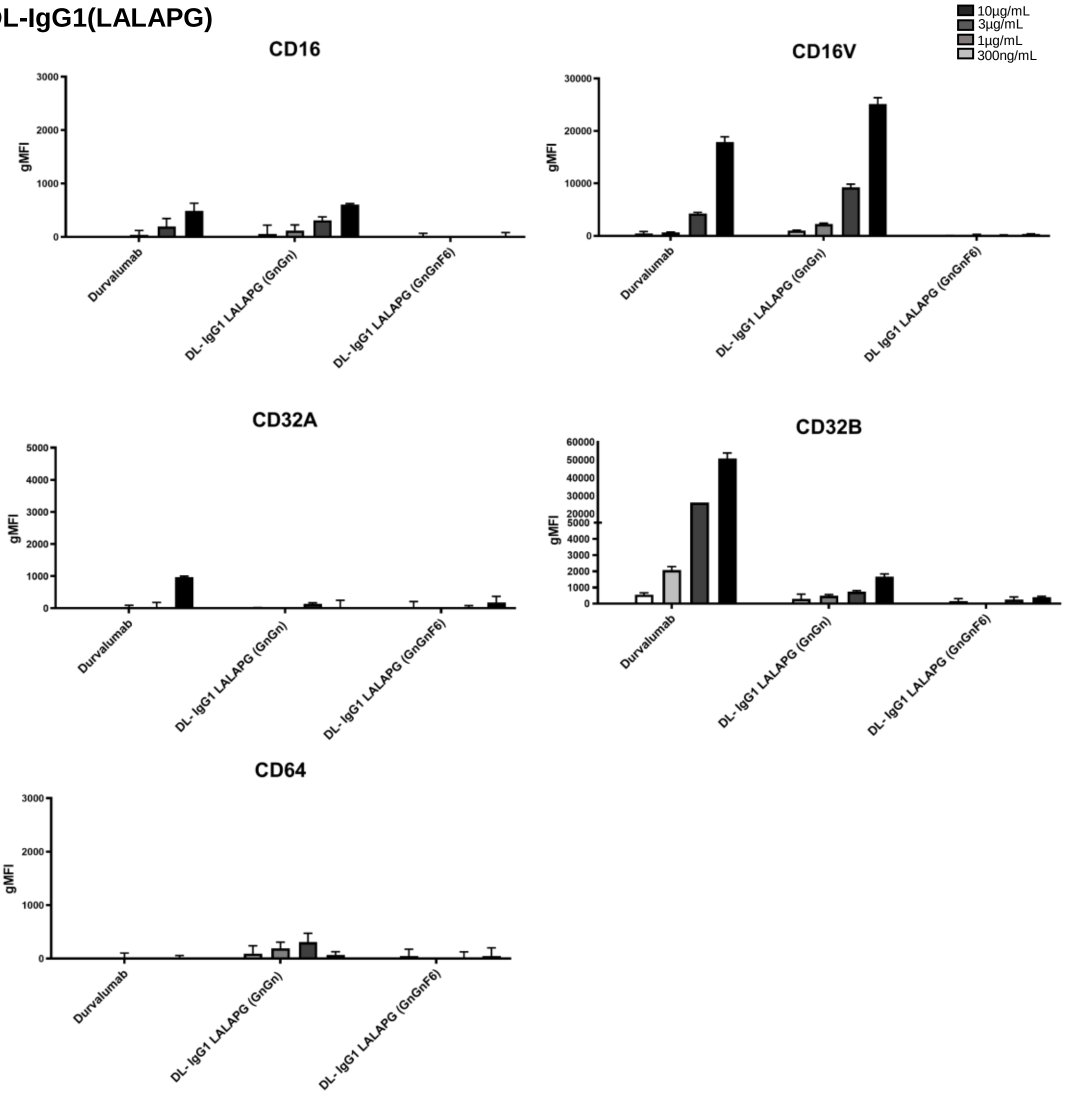

Figure S4 continued

C. DL-IgG4(PVA)$_{S228P}$

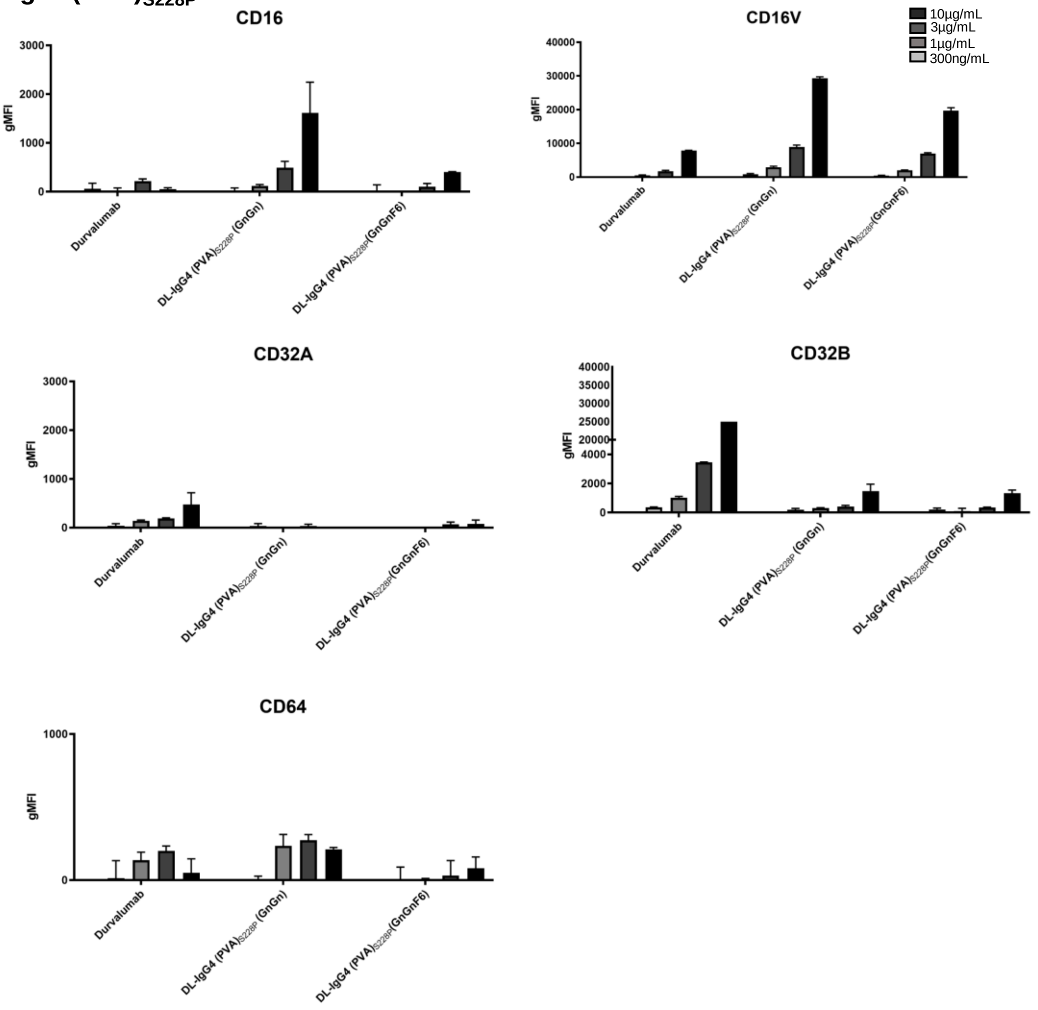

D. DL-IgG4(PVA)$_{S228P}$ vs. DL-IgG4$_{S228P}$

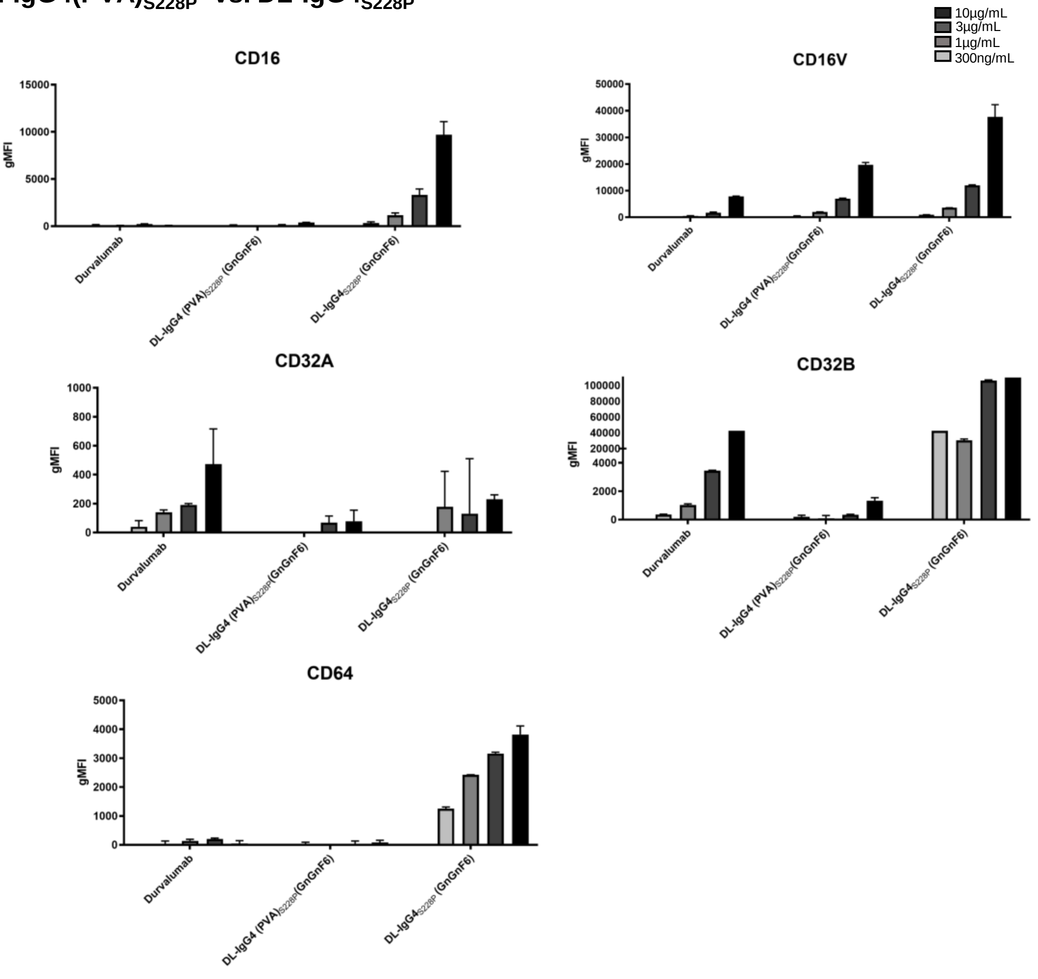

Figure S4: Binding of Durvalumab variants to Fcγ receptors

Binding of afucosylated (GnGn) and fucosylated (GnGnF6) glyco-variants of plant-derived DL to cells expressing CD16, CD16V, CD32A, CD32B and CD64 Fcγ receptors was compared to Durvalumab (Imfinzi®): (**A**) DL-IgG1 (**B**) DL-IgG1(LALAPG) (**C**) DL-IgG4(PVA)$_{S288P}$ (**D**) Comparison of binding of fucosylated (GnGnF6) DL-IgG4(PVA)$_{S288P}$ and DL-IgG4$_{S228P}$. Cells not expressing human Fcγ receptors served as control to normalize data.

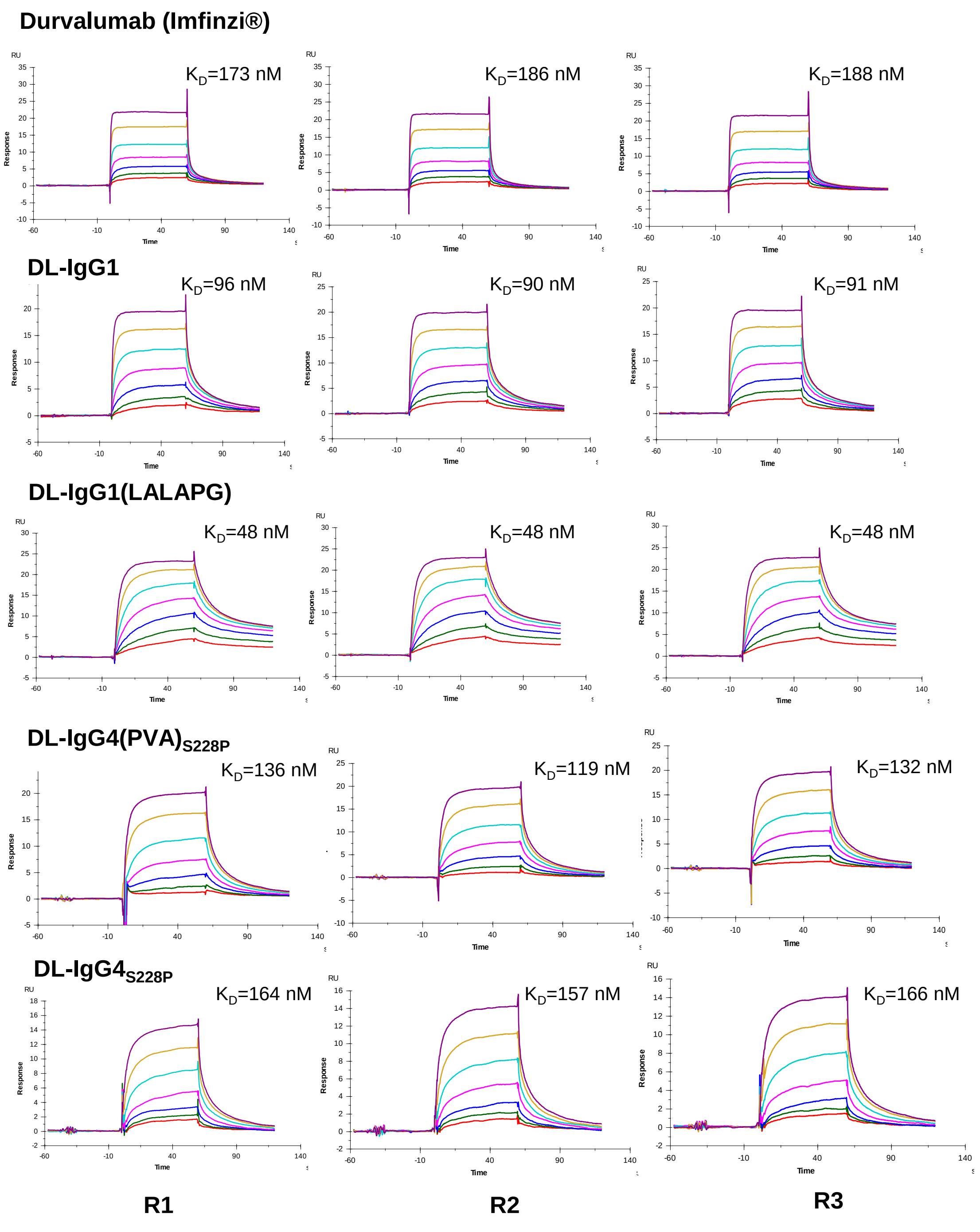

Figure S5: SPR sensograms for the binding of Durvalumab variants to hFcRn

SPR sensograms showing the binding of Durvalumab variants to hFcRn. Experiments were done in three runs (R1-R3) using DL concentrations of: 6.87, 13.75, 27.5, 50.5, 110, 220 and 440 nM. Running buffer (blank) was applied before and after each experiment and was subtracted from the binding response. K_D values are given for each sensogram (see Table S1).

Table S1

Antibody	Run	kD (nM)	Rmax (RU)
DL-IgG1	1	96	22.8
	2	90	22.2
	3	91	21.5
DL-IgG1(LALAPG)	1	48	23.9
	2	48	23.8
	3	48	23.5
DL-IgG4(PVA) $_{S228P}$	1	136	26.2
	2	119	25.5
	3	132	25.2
DL-IgG4$_{S228P}$	1	164	18.9
	2	157	18.3
	3	166	18.6
Durvalumab (Imfinzi®)	1	173	27.6
	2	186	27.9
	3	188	27.9

Table S1: Kinetic parameters of the binding of Durvalumab variants to hFcRn
Dissociation equilibrium constants (K_D) and binding responses (Rmax) to assess affinity of plant-derived fucosylated DL variants to human FcRn receptor compared to Durvalumab (Imfinzi®). Values are given for each antibody analyzed in three separate runs. RU, response unit.
