# Supplementary figures and images for: Plant‐derived Durvalumab variants show efficient PD‐1/PD‐L1 blockade and therapeutically favourable FcR binding
Source: Plant Biotechnol J. 2023 Dec 4;22(5):1224–37. doi: 10.1111/pbi.14260 (PMC11022803; doi:10.1111/pbi.14260)

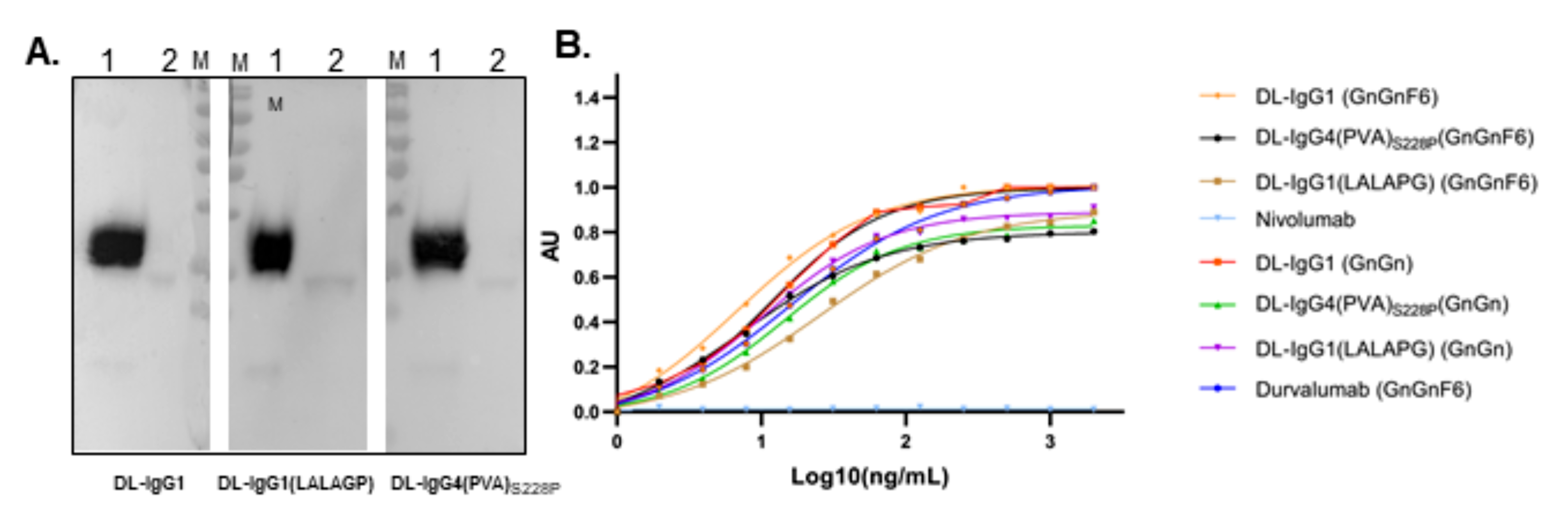

Supplement: Supplementary file 1 — Figure S1 Binding of Durvalumab variants to PD‐L1His. Figure S2 Expression, purification and glycosylation of plant‐derived PD‐L1His. Figure S3 Blocking PD‐1/PD‐L1 interaction by Durvalumab. Figure S4 Binding of Durvalumab variants to Fcγ receptors. Figure S5 SPR sensorgrams for the binding of Durvalumab variants to hFcRn. Table S1 Kinetic parameters of the binding of Durvalumab variants to hFcRn. [file PBI-22-1224-s001.zip › Fifure S2 new.jpg]
