# Supplementary material for: Plant‐derived Durvalumab variants show efficient PD‐1/PD‐L1 blockade and therapeutically favourable FcR binding
Source: Plant Biotechnol J. 2023 Dec 4;22(5):1224–37. doi: 10.1111/pbi.14260 (PMC11022803; doi:10.1111/pbi.14260)
Supplement: Supplementary file 2 — Appendix S1 Transient expression‐agroinfiltration. [file PBI-22-1224-s002.pdf]

**Izadi et al.: Supplementary methods**

Transient expression-agroinfiltration

*N. benthamiana* glycosylation mutant plants (ΔXF, Strasser et al., 2008) were grown at 22°C with a 16-h light/ 8-h dark photoperiod. Four to five-week-old plants were used for agroinfiltration experiments. Liquid cultures of agrobacteria transformed with plasmids for the expression of Durvalumab heavy and light chains and for PD-$L1_{His}$ were grown at 29 °C for 24 h. Cells were harvest by centrifugation at 3000 g for 4 min and resuspended in infiltration buffer (10 mM MES pH 5.6; 10 mM MgSO4). Bacterial cultures used to express Durvalumab (HC and LC) and PD-$L1_{His}$ were infiltrated at an optical density ($OD_{600}$) of 0.3 while FUT8 (Castilho et al., 2015) was infiltrated at $OD_{600}$ of 0.1. Infiltrated leaves were harvested four days post infiltration.

Apoplastic fluid collection

To collect proteins secreted into the apoplastic fluid (AF), infiltrated leaves were immersed in buffer solution (20 mM $Na_2HPO_4$, 100 mM NaCl, pH 7.4) and subjected to vacuum (2×5 min). After blotting dry with filter paper, leaves were centrifuged (1000 rpm for 15 min) in 50 mL tubes, containing a supported mesh which allows for the separation and recovery of the AF from the leaf material (Castilho and Steinkellner, 2016). After filtration through a 0.45 µm membrane filter (Merck Millipore), NaCl and imidazole were added to adjust the buffer to 20 mM $Na_2HPO_4$, 500 mM NaCl, and 10 mM imidazole (pH 7.4). On average, one gram of leaf material renders approximately 650µL of AF.

Total soluble protein extraction

Infiltrated leaves were used to extract total soluble proteins. Briefly, infiltrated plant leaf material (~30 g) was ground using a kitchen mixer with 4 mL/g of extraction buffer (50 mM Tris/HCl, 150 mM NaCl, supplemented with 40 mM ascorbic acid and 4 % PVPP, pH 7.4) at 4 °C. The slurry was incubated for 30 min at 4 °C, centrifuged (11 000 rpm for 20 min at 4°C) and filtered through 2 layers of paper filters. The filtrate was further clarified by a pH shift at 4 °C, by adding 5 M HCl till pH 5, then stirred on ice for 20 min and prior to pH adjustment to 7.0 with 5 M NaOH. Next, samples were centrifuged twice at 11 000 rpm for 20 min and at 18 000 rpm for 30 min. The final supernatant was stored over night at -80°C. Finally, after 40 min centrifugation at 18 000 rpm the extract was filtered through a 0.22 µm filter.

Enzyme-Linked Immunosorbent Assays (ELISA)

ELISA conditions were first optimized in terms of PD-$L1_{His}$ coating conditions (50-200 ng/mL) and DL-dilutions (5000 ng- 2 ng).

Wells of 96-well microplates (Thermofisher Maxisorp, M9410-1CS) were coated with 200 ng of plant-derived PD-$L1_{His}$, overnight at 4°C. Plates were blocked with 100µL 3% BSA in PBS-T (PBS with 0.05% Tween 20) for 1.5 h, washed and incubated for 2 h with serial dilutions of Durvalumab (starting from 2 µg/mL). Anti-human IgG conjugated to horseradish peroxidase (Promega, 1:20 000 in PBS-T) was added and incubated for 1 h at room temperature. The plates were developed using 50 µL/well 3,3',5,5'-tetramethylbenzidine (Sigma) for 5 min and the reaction was stopped with 50µL/well of 2M H2SO4. Absorbance was measured at 450 nm with reference to 620 nm using a Tecan Spark® spectrophotometer. Effective half-maximum concentrations ($EC_{50}$) were defined at the infliction point of the sigmoidal curve by non-linear regression of the blank-corrected absorbance values using GraphPad Prism (version 8).
Durvalumab (Imfinzi®, AstraZeneca) and Nivolumab (Opdivo®, Bristol-Myers Squibb) were used as controls.

Gastrointestinal cancer cell lines and treatment

Colorectal (SW48) and gastric (NCI-N87) cancer cell lines were purchased from the American Type Culture Collection (Manassas, VA, USA) and cultured at 37°C in a 5% $CO_2$ atmosphere. Both cell lines were cultured in RPMI-1640 GlutaMAX$^{TM}$, HEPES medium (Biowest, Riverside, MO, USA), supplemented with 10% heat-inactivated fetal bovine serum (FBS) (Biowest). All cell lines had their identity confirmed by short tandem repeat (STR) profiling by the PowerPlex® 16 HS System kit (Promega, Madison, WI, USA), and were routinely tested for mycoplasma contamination by PCR amplification. To induce PD-L1 expression, cell lines were seeded subconfluently (500.000 cells/well) in 6-well plates and stimulated with 40ng/ml IFNγ (Immunotools, Friesoythe, Germany) for 48 h. After 48 h, cells were harvested, washed with PBS containing 1% BSA, and stained with 1µg/mL of Durvalumab (Imfinzi®) and respective plant-derived variants for 30 min on ice, followed by incubation with secondary anti-human Alexa Fluor$^{TM}$ 488-conjugated antibody (Invitrogen$^{TM}$, ThermoFisher Scientific, Waltham, MA, USA). Prior to flow cytometry, 15 µL of 2 µg/mL of DAPI (Sigma-Aldrich) was added to cells, and viability was assessed as the frequency of DAPI-negative cells. Signals were acquired using a LSRFortessa system (BD Bioscience, San Jose, CA, USA) and data was analyzed using FlowJo software (BD Bioscience).

Cell reporter lines culture and flow cytometry

The Jurkat (JE6.1) cell line and the human erythroleukemia K562 cell line were derived from in house stocks. JE6.1 reporter cells expressing an NF-kB::eGFP reporter gene and high levels of

human PD-1 (JE6.1-NF-kB::eGFP-PD1) were described previously (De Sousa Linhares et al., 2019; Jutz et al., 2017). The K562-based stimulator (K562S) cells, which stably express a construct encoding a membrane-bound human CD3 Ab single-chain fragment (CD14 stem) have been described previously (Battin et al., 2022). For this study we generated K562S cells expressing high levels of human PD-L1. Both cell lines were cultured in RPMI1640 supplemented with 10% FBS, penicillin (100 U/mL) and streptomycin (100 µg/mL) (all from Sigma- Aldrich). All cells were routinely tested for mycoplasma contaminations using a previously reported method (Battin et al., 2017). Flow cytometry was performed using a Cytoflex-S flow-cytometer (Beckman-Coulter) equipped with a plate reader. FlowJo software (version 10.4.1. Tree Star) was used for flow cytometry analysis.

**Supplementary References**


Battin, C., Hennig, A., Mayrhofer, P., Kunert, R., Zlabinger, G.J., Steinberger, P., Paster, W., (2017) A human monocytic NF-kappaB fluorescent reporter cell line for detection of microbial contaminants in biological samples. Plos One 12, e0178220.

Battin, C., Kaufmann, G., Leitner, J., Tobias, J., Wiedermann, U., Rolle, A., Meyer, M., Momburg, F., Steinberger, P., (2022) NKG2A-checkpoint inhibition and its blockade critically depends on peptides presented by its ligand HLA-E. Immunology 166, 507-521.

Castilho, A., Gruber, C., Thader, A., Oostenbrink, C., Pechlaner, M., Steinkellner, H., Altmann, F., (2015) Processing of complex N-glycans in IgG Fc-region is affected by core fucosylation. MAbs 7, 863-870.

Castilho, A., Steinkellner, H., (2016) Transient Expression of Mammalian Genes in N. benthamiana to Modulate N-Glycosylation. Methods Mol Biol 1385, 99-113.

De Sousa Linhares, A., Battin, C., Jutz, S., Leitner, J., Hafner, C., Tobias, J., Wiedermann, U., Kundi, M., Zlabinger, G.J., Grabmeier-Pfistershammer, K., Steinberger, P., (2019) Therapeutic PD-L1 antibodies are more effective than PD-1 antibodies in blocking PD-1/PD-L1 signaling. Sci Rep 9, 11472.

Jutz, S., Hennig, A., Paster, W., Asrak, O., Dijanovic, D., Kellner, F., Pickl, W.F., Huppa, J.B., Leitner, J., Steinberger, P., (2017) A cellular platform for the evaluation of immune checkpoint molecules. Oncotarget 8, 64892-64906.

Strasser, R., Stadlmann, J., Schahs, M., Stiegler, G., Quendler, H., Mach, L., Glossl, J., Weterings, K., Pabst, M., Steinkellner, H., (2008) Generation of glyco-engineered Nicotiana benthamiana for the production of monoclonal antibodies with a homogeneous human-like N-glycan structure. Plant Biotechnol J 6, 392-402.
